# A systematic dissection of the epigenomic heterogeneity of lung adenocarcinoma reveals two different subclasses with distinct prognosis and core regulatory networks

**DOI:** 10.1186/s13059-021-02376-1

**Published:** 2021-05-17

**Authors:** Chongze Yuan, Haojie Chen, Shiqi Tu, Hsin-Yi Huang, Yunjian Pan, Xiuqi Gui, Muyu Kuang, Xuxia Shen, Qiang Zheng, Yang Zhang, Chao Cheng, Hui Hong, Xiaoting Tao, Yizhou Peng, Xingxin Yao, Feilong Meng, Hongbin Ji, Zhen Shao, Yihua Sun

**Affiliations:** 1 Department of Thoracic Surgery and State Key Laboratory of Genetic Engineering, Fudan University Shanghai Cancer Center, Shanghai, 200032 China; 2 Institute of Thoracic Oncology, Fudan University, Shanghai, 200032 China; 3 Department of Oncology, Shanghai Medical College, Fudan University, Shanghai, 200032 China; 4 CAS Key Laboratory of Computational Biology, Shanghai Institute of Nutrition and Health, University of Chinese Academy of Sciences, Chinese Academy of Sciences, Shanghai, 200031 China; 5 State Key Laboratory of Cell Biology, Shanghai Institute of Biochemistry and Cell Biology, Center for Excellence in Molecular Cell Science, Chinese Academy of Sciences, Shanghai, 200031 China; 6 Department of Pathology, Fudan University Shanghai Cancer Center, Shanghai, 200032 China; 7 State Key Laboratory of Molecular Biology, Shanghai Institute of Biochemistry and Cell Biology, Center for Excellence in Molecular Cell Science, Chinese Academy of Sciences, Shanghai, 200031 China; 8 School of Life Science and Technology, Shanghai Tech University, Shanghai, 200120 China

**Keywords:** Lung adenocarcinoma, Classification model, Epigenome, Super-enhancers, Core regulators

## Abstract

**Background:**

Lung adenocarcinoma (LUAD) is a highly malignant and heterogeneous tumor that involves various oncogenic genetic alterations. Epigenetic processes play important roles in lung cancer development. However, the variation in enhancer and super-enhancer landscapes of LUAD patients remains largely unknown. To provide an in-depth understanding of the epigenomic heterogeneity of LUAD, we investigate the H3K27ac histone modification profiles of tumors and adjacent normal lung tissues from 42 LUAD patients and explore the role of epigenetic alterations in LUAD progression.

**Results:**

A high intertumoral epigenetic heterogeneity is observed across the LUAD H3K27ac profiles. We quantitatively model the intertumoral variability of H3K27ac levels at proximal gene promoters and distal enhancers and propose a new epigenetic classification of LUAD patients. Our classification defines two LUAD subgroups which are highly related to histological subtypes. Group II patients have significantly worse prognosis than group I, which is further confirmed in the public TCGA-LUAD cohort. Differential RNA-seq analysis between group I and group II groups reveals that those genes upregulated in group II group tend to promote cell proliferation and induce cell de-differentiation. We construct the gene co-expression networks and identify group-specific core regulators. Most of these core regulators are linked with group-specific regulatory elements, such as super-enhancers. We further show that CLU is regulated by 3 group I-specific core regulators and works as a novel tumor suppressor in LUAD.

**Conclusions:**

Our study systematically characterizes the epigenetic alterations during LUAD progression and provides a new classification model that is helpful for predicting patient prognosis.

**Supplementary Information:**

The online version contains supplementary material available at 10.1186/s13059-021-02376-1.

## Background

Lung cancer is a malignant tumor with highest mortality among all cancers worldwide and could be further classified into small cell lung cancer (SCLC) and non-small cell lung cancer (NSCLC) [1]. Lung cancer exhibits high heterogeneity that enables adaptability, limits therapeutic success, and remains incompletely understood. Lung adenocarcinoma (LUAD), which accounts for most cases of NSCLC, can be classified into several histologic subtypes based on morphological characters [2]. These subtypes are frequently associated with patient prognosis, e.g., the micropapillary and solid predominant subtypes are predictors of poor prognosis [3, 4]. The histologic classification is most widely used in clinical practice but is also limited since it is a subjective judgment by pathologists. Other classification methods like radiological classification can also predict prognosis [5, 6], and with the development of targeted therapy and precision medicine, molecular classification is becoming more and more important [7].

Targeted therapies focusing on *EGFR* mutation, *ALK*-fusion, and *ROS1*-fusion have dramatically improved LUAD patient survival [8–10]. Although LUAD is one of the most heavily mutated cancers, most mutations, such as *TP53*, *KRAS,* and *STK11,* are not pharmaceutically targetable [11]. *EGFR* mutation is one of the most common driver genes, but can only explain about 14% of Caucasian LUAD patients and 50% of East-Asian patients [7, 12]. As a consequence, many patients are lacking therapeutic targets and need to be further characterized. Another important molecular classification is based on transcriptomic signatures. Transcriptomic classification not only defines distinct LUAD subclasses, but also reveals their relation with prognosis, critical biological features, and potential oncogenes [7, 13–15].

The study of cancer epigenetics has radically altered our views in cancer pathogenesis, providing new insights in biomarker development for risk assessment, early detection, and therapeutic stratification [16–19]. DNA methylation profiling of tumor tissues divides LUAD into distinct subsets: significantly altered CpG island methylator phenotype high (CIMP-H(igh)) group, normal-like CIMP-L(ow) group, and intermediate methylation group. Among them, DNA hypermethylation of several key genes, such like *CDKN2A*, *GATA2,* and *WIF1*, are observed in CIMP-H tumors [7]. On the other hand, histone modifications are also found to play important roles in LUAD [20–23]. The histone modification status is closely connected with chromatin configuration and *cis* element activity. However, it is also variable in tumors as it can be easily influenced by abnormal genomic rearrangements, mutations, and histone modification enzymes [24]. Recently, the Encyclopedia of DNA Elements (ENCODE) and the Roadmap Epigenomics Consortiums have extensively characterized human regulatory landscape across a wide range of cell lines and tissues, expanding our understanding of cancer regulatory abnormalities [25, 26]. Although histone modifications are widely recognized as key epigenetic regulators, rare study has been performed to profile and understand the dynamic histone modification patterns in primary tumor tissues of LUAD patients.

H3K27ac is closely linked with gene transcriptional activation and acts as a major histone mark of active regulatory elements, especially for enhancers and super-enhancers (SEs). In our previous study, we investigated H3K27ac landscape in two Chinese patient-derived LUAD cell lines and revealed SE-associated gene *RAI14* as a novel biomarker [27]. Here, we aim to further explore the dynamic H3K27ac landscape in LUAD tumors. We performed high-resolution chromatin immunoprecipitation sequencing (ChIP-seq) of H3K27ac in the tumors and normal lung tissues of 42 LUAD patients and uncovered a high intertumoral epigenetic heterogeneity. We classified the LUAD patients into two major subgroups based on the observed epigenetic heterogeneity and found they have distinct prognosis and transcriptomic features. Through an extensive co-expression network analysis, we defined the core transcriptional and epigenetic regulators for each subgroup, and identified *CLU* as a novel tumor suppressor from the downstream target genes of these core regulators in LUAD. Taken together, our study expands the understanding of LUAD complexity by a systematic analysis of epigenetic and transcriptomic signatures, providing important supplement to current histologic and molecular classifications.

## Results

### The epigenetic landscape differed between normal lung and LUAD tissues

To explore the epigenetic landscape in LUAD, we assembled a cohort of clinical samples from patients who received surgery in Fudan University Shanghai Cancer Center (FUSCC). The histological subtype and tumor cell content of each LUAD sample were confirmed by two independent pathologists. H3K27ac high-throughput chromatin immunoprecipitation sequencing (ChIP-seq) was then successfully performed in 42 paired LUAD and adjacent normal lung samples. Among the 42 LUAD samples, 1 sample was minimally invasive adenocarcinoma (MIA) and 41 samples were invasive adenocarcinomas (IACs) containing different subtypes (Additional file 2: Table S1).

**Fig. 1:**
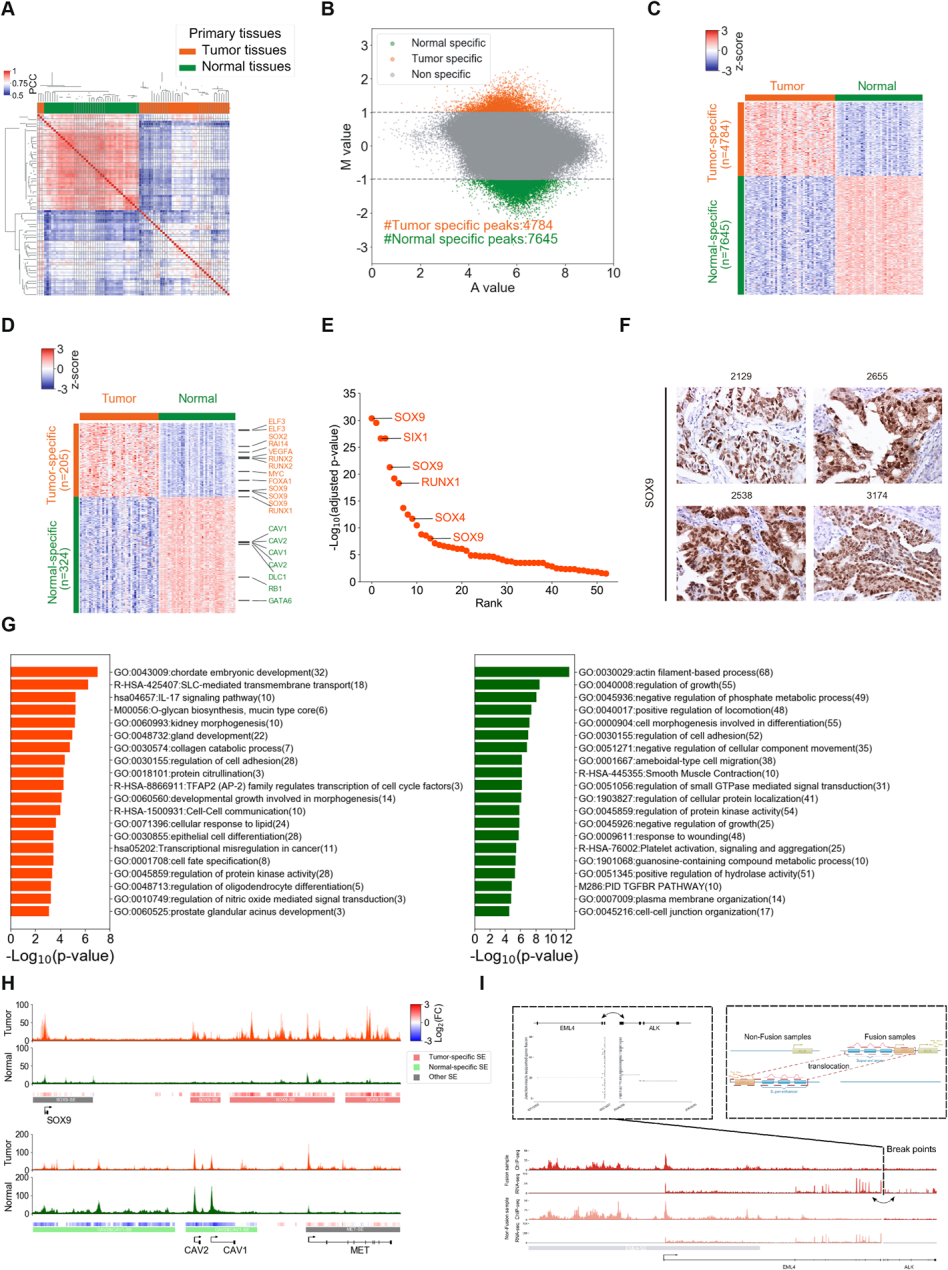
Distinct H3K27ac profiles in LUAD and normal lung tissues. **a** Unsupervised hierarchical clustering of H3K27ac profiles for tumor and normal lung tissues of LUAD patients based on pairwise Pearson correlation coefficients (PCCs). **b** An MA plot of differential H3K27ac-modified sites between tumor and normal tissues, “M” represented log 2 fold change and “A” represented average log 2 signal intensities, sites with |M value| ≥ 1 and adjust *p*-value ≤ 0.05 defined as differential sites. **c** A heatmap of the H3K27ac signal in differential sites identified in **b**. The H3K27ac signal is represented as row-normalized z-scores. **d** Differential H3K27ac enrichment in super-enhancers (SE) between tumor and normal tissues. Each row represents an SE with a different enrichment between two tissues. SE scores are represented as row-normalized z-scores. Important differential SE-associated genes shown in the right. **e** Ranked plot for tumor-specific SE-associated TFs. IHC staining validated TFs are indicated with lines. **f** IHC staining results of 4 tumor samples showed tumor-specific SE-associated *SOX9* were highly expressed in tumor. **g** The functional enrichment of tumor-specific (left) and normal-specific (right) SE-associated genes. **h** Track plots of the H3K27ac signal distribution in tumor (top) and normal (bottom) samples across the *SOX9* (tumor-specific super-enhancer associated genes), *CAV1-CAV2* (normal-specific super-enhancer associated genes), and *MET* (other super-enhancer associated genes) loci. “SOX9-SE” represented this super-enhancer associated with *SOX9*. “CAV2&CAV1-SE” represented this super-enhancer associated with *CAV2* and *CAV1*. “MET-SE” represented this super-enhancer associated with *MET*. Heatmap of log2 fold change indicates the H3K27ac signal differences between tumor and normal tissues. **i** An example of super-enhancer hijacking. Number of junction reads from RNA-seq supported *EML4* and *ALK* gene fusion showed in the top left panel. Model of super-enhancer hijacking through chromosome translocation showed in the top right panel. Track plots of the H3K27ac signal distribution and gene expression in fusion and non-fusion samples across the *EML4* and *ALK* loci (bottom)

We next processed the ChIP-seq data for each sample as previously described before further analysis [28, 29]. Briefly, after data quality control, reads alignment, peak calling, and filtering, in total we identified ~ 100 thousand H3K27ac peaks for LUAD samples and ~ 60 thousand peaks for normal samples, mostly located at intergenic and intronic regions (Additional file 1: Figures S1A and S1C). Interestingly, the LUAD and normal lung tissues can be well separated by their H3K27ac landscape, indicating that epigenetic alteration was one of the major differences and might play important roles in LUAD tumorigenesis (Fig. 1a, Additional file 1: Figure S1B). We grouped the H3K27ac ChIP-seq profiles of LUAD and normal lung samples, and then used the MAnorm2 model to compare these two groups of profiles [29]. By this method, we detected a huge number of differential H3K27ac sites, including 4784 tumor-specific and 7645 normal-specific ones (Fig. 1b, c). We found that genes regulated by the normal-specific sites were enriched for basic cellular functions, such as actin cytoskeleton organization and GTPase activity whereas the tumor-specific sites were significantly associated with a number of genes known to be dysregulated in cancers (Additional file 1: Figure S1D), indicating that aberrant epigenetic modifications directly contribute to transcriptional dysregulation in LUAD.

We and others have previously investigated the H3K27ac landscape in cell lines instead of tissue samples to explore LUAD epigenetic alterations [27, 30]. Strikingly, we found that the H3K27ac profiles of LUAD cell lines, including the widely used A549 cell line and two derived from Chinese patients, were quite different from those of primary tumor tissues (Additional file 1: Figure S1E). This indicated that observation obtained from cell lines may not accurately represent the in vivo situation and directly profiling the epigenetic landscape in primary tissues is of irreplaceable value to cancer studies. Taken together, these data uncovered the dramatic difference of the H3K27ac landscape between LUAD and normal lung tissues, which might be of functional importance.

### Super-enhancers control LUAD driver oncogenes

Previous studies have revealed that large clusters of enhancers, named super-enhancers (SEs), have prominent roles in determining cell identity, tumorigenesis, and chronic disease [28]. Compared to typical enhancers (TEs), SEs elicit stronger effects and predominantly exert a transactivation function to induce continuous and high expression of target genes [31]. We thus wanted to identify the SEs in our dataset and determine the role of SEs in LUAD tumorigenesis. To do so, we first separately identified SEs for each H3K27ac ChIP-seq sample using ROSE, a software specifically developed for this purpose [31, 32]. By this means, we identified ~ 800 and ~ 700 SEs for each tumor and normal sample, respectively, leaving the other distal H3K27ac sites as TEs (Additional file 1: Figure S1F). The SEs only accounted for a minor portion of the total enhancer domains (median, 5.58%) but for the majority of H3K27ac signals (median, 33.76%) (Additional file 1: Figure S1G). We merged the SEs identified from all tumors and normal samples and obtained 2,893 SEs in total. We further mapped the differential H3K27ac sites detected between LUAD and normal lung samples to these SEs and thus defined 205 tumor-specific and 324 normal-specific SEs (Fig. 1d; Additional file 1: Figure S2A - S2C). Interestingly, > 50% of the 11,574 differential H3K27ac sites at distal regions were located within SEs (Additional file 1: Figure S2D). This finding emphasizes that SE abnormalities might play an important role in LUAD development.

Generally, SEs and TEs regulate spatially closed genes, but they can also contact distant gene transcription start sites (TSSs) by forming long-range enhancer-promoter interactions [33]. While the most widely used method for annotating the target gene of each regulatory element is to map it to gene with the nearest TSS, we used a more reliable gene annotation method here which considered the correlation between the normalized H3K27ac intensities of each SE and those at gene promoters within 500 kb of the SE across all tumors and normal samples (Additional file 1: Figure S3A - 3C showing the SE at *MAX*-*FUT8* gene locus as an example). In this way, we found that a number of well-known oncogenes, including *MYC* and *SOX9*, were annotated as targets of tumor-specific SEs, while several tumor suppressor genes in LUAD, such as *DLC1* and *RB1*, were linked to normal-specific SEs (Fig. 1d; Additional file 3: Table S2, Additional file 4: Table S3). Among tumor-specific SE-associated genes, lots of genes were important transcriptional factors (TFs). Then, we ranked all the TFs and found *SOX9* was ranked as top one tumor-specific SE-associated TF (Fig. 1e). We performed immunohistochemistry (IHC) staining of tumor-specific SE-associated transcription factor *SOX9* on patient tissue samples and found that higher level of SOX9 was observed in tumors than adjacent normal lung tissues (Fig. 1f). We also performed IHC staining of other top-ranked TFs including *SIX1*, *RUNX1,* and *SOX4* and again found higher expression level of these TFs in tumor tissues (Additional file 1: Figure S3E). The tumor-specific SE-associated genes were enriched in important pathways, such as chordate embryonic development (*RUNX2, FOXA1*), regulation of cell adhesion (*RUNX1, SOX2* and *VEGFA*), and epithelial cell differentiation (*SOX9, ELF3*) (Fig. 1g). Some of these pathways are related to cancer stem cell (CSC) or LUAD cancer cell migration and invasion, including chordate embryonic development, cell fate specification, regulation of cell adhesion, and collagen catabolic process [34, 35]. In particular, the activity of *SOX9* was known to be associated with the primitive transcriptional programs spanning stem cell-like to regenerative pulmonary epithelial progenitor states during metastasis in LUAD, consistent with a lot of epithelial differentiation and embryonic related pathways enriched in tumor-specific super-enhancer associated genes [34]. Genes associated with normal-specific SEs were enriched in cell growth regulation (*DLC1*, *RB1*, *GATA6*) and other basic cellular functions, such as cell morphogenesis, GTPase-mediated signal transduction and regulation of phosphate metabolic process (Fig. 1g). For example, *CAV1* and *CAV2* were marker genes of lung alveolar cells [36], and the H3K27ac levels at their nearby SEs were significantly upregulated in normal lung tissues compared to LUAD (Fig. 1h). Thus, in both normal lung and LUAD samples, SEs regulated key cell identity genes, and the SE abnormality contributed to tumorigenesis.

Previous studies have revealed that genomic rearrangement is highly associated with SE abnormality in cancers, and thus we focused on the most frequent fused oncogene *ALK* in LUAD [37]. *ALK*-fusion is a powerful driver mutant and important therapeutic target that occurs in ~ 5% of LUAD patients, especially in advanced stage patients [8]. The malignant behavior caused by *ALK*-fusion can be strongly inhibited by receptor tyrosine kinase inhibitors (TKIs), thus dramatically improved patients’ prognosis [38]. Three of the 42 patients involved in our study were detected with *ALK*-*EML4* gene fusion, which hijacked (or translocated) the SE located upstream of *EML4* (Fig. 1i). It was suggested that the fusion protein can activate several canonical signaling pathways, including RAS/MEK/ERK pathway and PI3K/AKT cascades [39], and our results indicated that the hijacked SE probably maintained aberrant expression of *ALK-EML4*. Similar SE hijacking was also found in one patient with *ROS1-SLC34A2* fusion (Additional file 1: Figure S3F). These data suggested that SE abnormality could also directly get involved in LUAD oncogenesis through SE hijacking of driver oncogenes, and thus, the hijacked SE might be a potential target for LUAD therapy, especially for TKIs-resistant patients [32].

### LUAD is composed of two different epigenetic states

**Fig. 2:**
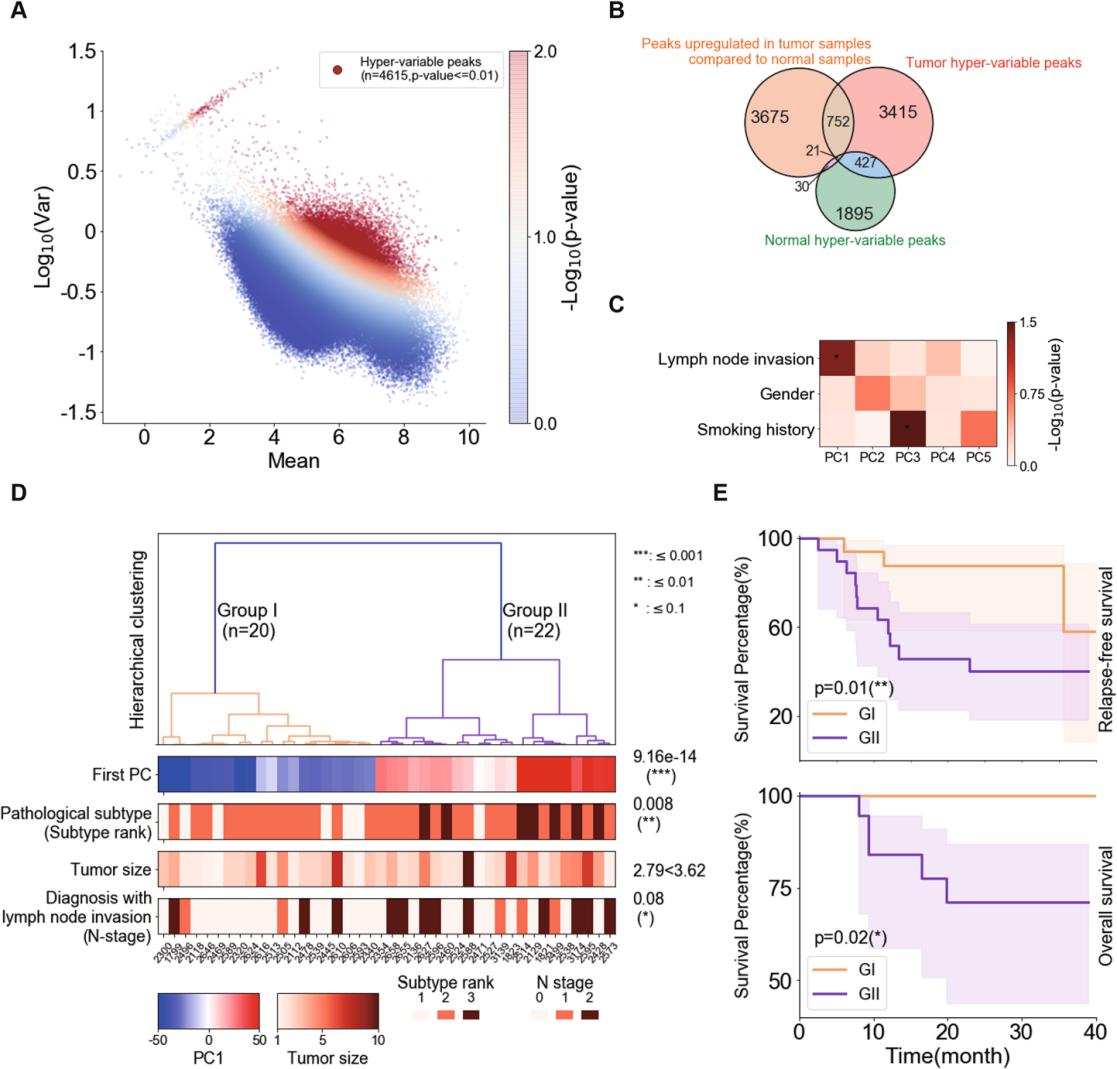
Epigenetic heterogeneity differentiates LUAD patient clinical outcomes. **a** The hyper-variable peaks (HVPs) identified based on the global trend of means and variances. The dots are colored according to the significance of the variance test performed by MAnorm2. Variable peaks with *p*-value less to 0.01 were defined as tumor hyper-variable peaks. **b** Venn diagram showed overlap between tumor hyper-variable peaks (hyper-variable peaks identified in tumor samples), peaks upregulated in tumor samples compared to normal samples (previous identified tumor-specific peaks), and normal hyper-variable peaks (hyper-variable peaks identified in normal samples). **c** The first 5 significant principle components and their correlation with lymph node invasion, gender, and smoking history; asterisk represented significant association (*p*-value of ANOVA less to 0.05). **d** Unsupervised hierarchical clustering using PC1 from a PCA on hyper-variable peaks identifying two subgroups, group I and group II. The associations between clinical characteristics and subgroups showed in the bottom, the *p*-values of rank-sum test were indicated to show the significance of associations. **e** Survival analysis of the two subgroups: relapse-free survival, RFS (top) and over-all survival, OS (bottom), and *p*-value of log rank test showed in the plot

Our data thus far highlighted the key epigenetic differences between normal lung and LUAD tissues. However, a high epigenetic heterogeneity was also observed among LUAD tissues from different patients (Fig. 1a). We next quantitatively evaluated the intertumoral variations of H3K27ac signals normalized by MAnorm2 on genome scale. In order to remove the mean-variance dependence, we fitted a mean-variance curve and selected those peaks with significant deviations above the curve [29]. By this method, we identified 4615 hyper-variable H3K27ac peaks (HVPs) among the tumor samples (Fig. 2a). We downloaded the susceptible SNPs of lung cancer identified by a previous genome-wide association study (GWAS) [40] and found they were highly enriched in the HVPs of LUAD samples (Additional file 1: Figure S4B). This finding suggests that the fluctuations of H3K27ac levels at these HVPs might provide valuable insights in understanding the epigenetic heterogeneity of LUAD. We further noticed that the vast majority of the tumor HVPs were not identified as HVPs across normal samples (Fig. 2b; Additional file 1: Figure S4C). It indicated that most of the intertumoral epigenetic heterogeneity observed at the tumor HVPs could hardly be explained by the epigenetic variations already existed in the patients’ normal lung tissue and it predominantly emerged during tumorigenesis. Meanwhile, there was little overlap between the tumor HVPs and the peaks upregulated in tumor samples compared to normal samples (previous identified tumor-specific peaks) showing consistent H3K27ac changes between tumors and normal tissues (Fig. 2b), suggesting that a very large fraction of them may be peaks specific to a subset of tumor samples. Thus, further investigation on the observed intertumoral epigenetic heterogeneity was needed and might provide insights into LUAD progression.

We next performed principal component analysis (PCA) on the normalized H3K27ac intensities of tumor HVPs across all tumor ChIP-seq samples. Surprisingly, we found that, for each sample, its score on the first principle component (PC1) was significantly associated with the lymph node invasion status of corresponding patient (Fig. 2c). Hierarchical clustering of the tumor samples’ PC1 scores clustered them (and thus the corresponding patients) into two subgroups, namely group I and group II (GI and GII, Fig. 2d). Of note, LUAD can be classified into several subtypes which are important predictors of prognosis by its predominant histologic component [41–43]. Based on that, we have divided the 42 LUAD patients in our cohort into three pathological subgroups: low-risk, median-risk, and high-risk (Additional file 2: Table S1). Here, after a comprehensive comparison of the pathological subtype, tumor size, and lymph node metastasis status of patients in two subgroups, we observed a weak difference in tumor size and a significant difference in lymph node invasion between GI and GII patients (Fig. 2d). More importantly, we found that the tumor samples’ PC1 scores were strongly associated with the patient pathological subtypes and could largely distinguish the more aggressive tumors (GII) from the less aggressive ones (GI) (Fig. 2d). High-risk pathological subgroup composed of micropapillary and solid predominant LUAD tumors was only contained in GII patients (Additional file 2: Table S1). In contrast, GI contained more low-risk pathological subgroup patients (Fig. 2d, Additional file 2: Table S1). Survival analysis of these two groups of patients further supported that GII patients had a significantly poorer prognosis than GI patients (Fig. 2e). In summary, by systematically dissecting the inter-tumor epigenetic heterogeneity, we were able to classify the LUAD samples into two subgroups that correlated with clinical outcomes.

In addition to clinical features, we also compared the gene mutation patterns between GI and GII patients. *EGFR* mutation occurred in > 50% of Asian LUAD patients and was the most frequent driver mutation in our cohort (27 of all 42 patients). However, we found no difference in the *EGFR* mutation frequency between GI and GII (Additional file 1: Figure S4E). Our previous studies revealed that patients with *ALK*-fusion are more likely to be in advanced stages, and the *EML4-ALK* variant 3 usually indicates a poor prognosis [44]. Consistently, all 3 *ALK*-fusion positive patients were found in GII and had a solid predominant subtype (Additional file 1: Figure S4E). In conclusion, based on the intertumoral heterogeneity of H3K27ac profiles, we successfully classified LUAD into two distinct subgroups with different pathological subtypes and clinical outcomes.

### Epigenomic and transcriptomic changes underlie distinct pathways and malignancy between two LUAD subclasses

**Fig. 3:**
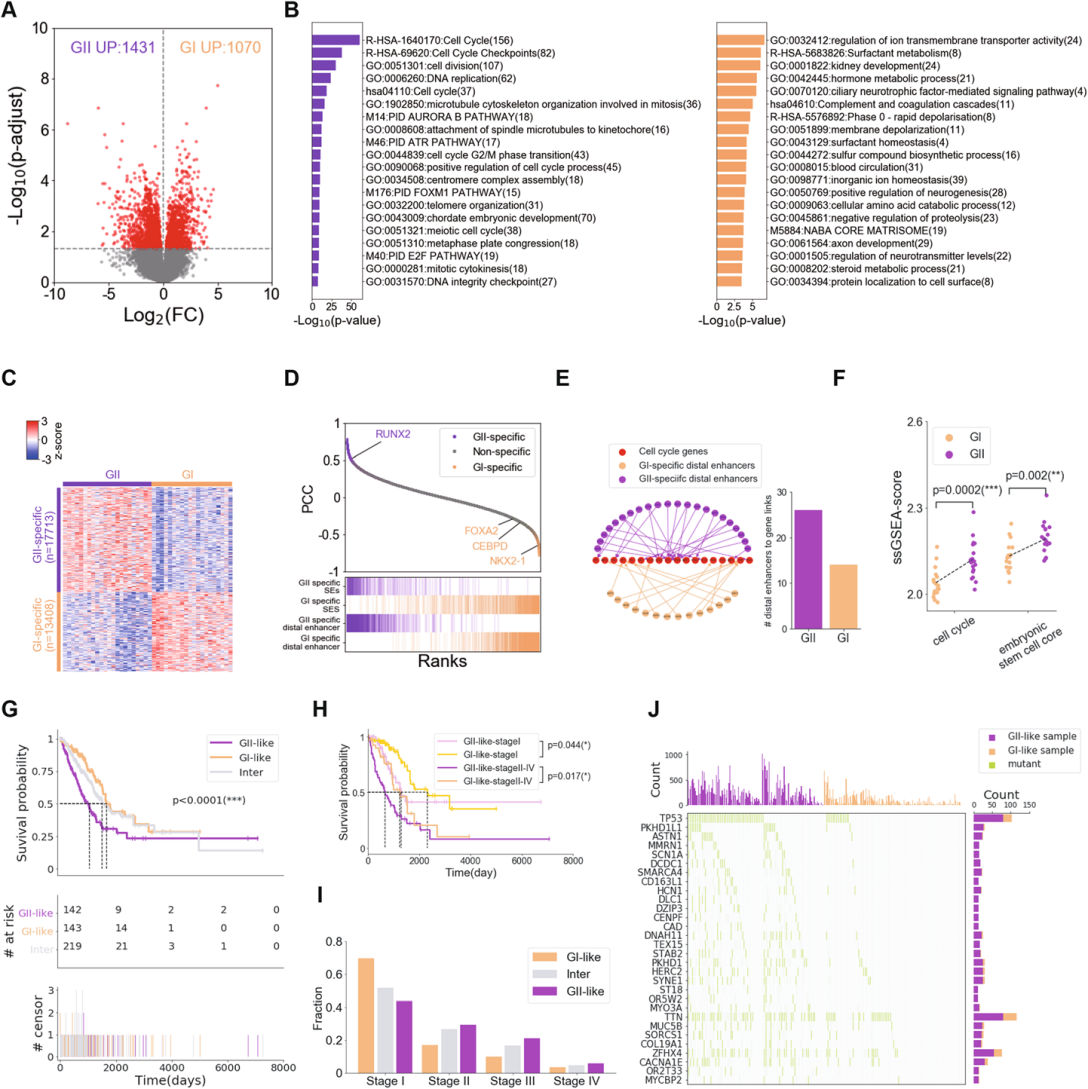
Transcriptomic and epigenetic alterations uncovered pathological pathways. **a** A volcano plot of the gene expression changes between group I (GI) and group II (GII). Genes with adjusted *p*-value less to 0.05 were defined as DEGs. **b** Functional enrichment of GII-specific (left) and GI-specific (right) genes. **c** A heatmap of the H3K27ac signals in group-specific peaks. The data were represented as row-normalized z-scores; each row represented a group-specific peaks, and each column represented a LUAD sample. **d** Genes ranked based on the correlation of gene expression and PC1 in hyper-variable peaks in tumor samples. The purple and orange bars (bottom) indicate GII-specific and GI-specific distal enhancers or SEs linked genes, respectively. **e** The convergence of GII-specific distal enhancers on cell cycle genes. **f** Comparison of ssGSEA-score of cell cycle pathway genes and embryonic stem cell core genes between GI and GII, t-test was performed between two different groups. **g** DEGs between GI and GII were used to group TCGA samples into GI-like, GII-like, and intermediate groups, and the K-M plot of patients’ survival in three groups, *p*-value determined by log rank test. ***p < 0.001. **h** The K-M plot of patients’ survival in GI-like and GII-like LUAD patients in stage I and stage II-IV, *p*-value determined by log rank test. *p < 0.05. **i** The distribution of GI-like, GII-like and intermediate samples across different tumor stages in the TCGA samples. **j** Top 30 bias somatic coding mutations in GI-like and GII-like LUAD patients. The middle panel showed somatic mutation by individuals (column) and by genes (row). The histogram on the top showed the number of mutations in each sample. The histogram on the right showed the differences in mutation frequency between GI-like and GII-like LUAD patients. Genes sorted by the *p*-value of Fisher-exact test

Our data suggested that patients in GI had better prognosis than patients in GII, as evidenced by the significant prolonged relapse-free survival (RFS) and over-all survival (OS) time (Fig. 2e). To investigate the reasons underlying these differences in survival, we performed RNA sequencing (RNA-seq) of the tumor samples and determined the differentially expressed gene (DEG) signatures for each group. In total, we identified 2501 significant DEGs between GII and GI (Fig. 3a). Functional enrichment analysis revealed that GII-upregulated genes were enriched in the cell cycle and DNA replication pathways, both of which were signatures of highly malignant tumors. Besides, *FOXM1* pathway and *E2F* pathway which were both enriched in GII have been proven to be related to LUAD invasion and metastasis [45, 46]. Conversely, a number of genes involved in maintenance of normal cell functions and metabolic processes were significantly downregulated from GI to GII (Fig. 3b). Consistent with GI-specific genes signatures, several independent studies have shown that *KRAS-*driven murine LUAD preferentially arises from alveolar type II (AT2) cells, supported by the IHC staining of alveolar markers in early-stage tumors [47]. Surfactant metabolism-related pathways were usually highly activated in AT2 cells but significantly decreased in GII compared to GI, indicating that GI might somehow represent early-stage tumors. Next, we moved to test the epigenomic differences between GI and GII. After a quantitative comparison of the H3K27ac profiles between GI and GII tumors using MAnorm2, we defined 17,713 GII-specific and 13,408 GI-specific H3K27ac peaks, which then led to the identification of 194 GII-specific and 437 GI-specific SEs. We further found that these SE were highly correlated with the genes differentially expressed between GI and GII (Fig. 3c, d; Additional file 1: Figures S6 and S7A). We chose two subgroup-specific SE-associated genes, *RUNX2* and *NKX2-1*(thyroid transcription factor 1, also known as *TTF1*), for IHC staining to assess their protein levels in GI and GII tumors. The LUAD maker *NKX2-1* was reported to suppress LUAD progression, whereas *RUNX2* was reported as key regulator to promote cancer progression [47, 48]. In our study, higher RUNX2 expression was detected in GII tumors compared to GI tumors, whereas NKX2-1 seemed to be specifically expressed in GI tumors (Additional file 1: Figure S7C and D).

To further investigate the significant pathways changed with epigenetic alterations, we ranked genes by the correlation of gene expression with PC1 scores across all tumor samples and performed gene set enrichment analysis (GSEA). The analysis confirmed the positive enrichment of gene sets related to cell cycle and DNA repair. On the contrary, the negative enrichment of genes set was associated with immune-cell signaling related pathway, such as asthma, complement, and coagulation cascades in aggressive tumors (Additional file 1: Figure S7B). These results indicated the strong connection between epigenetic alterations with transcriptional changes during LUAD progression. Accordingly, we found that more GII-specific enhancers were associated with cell cycle genes than GI-specific enhancers, indicating that epigenetic alterations might lead to group-specific transcription signatures (Fig. 3e; Additional file 1: Figure S8A).

We noticed that a number of lineage-specific TFs were upregulated in GI group and associated with GI-specific SEs, such as *NKX2-1* (LUAD marker), *FOXA2* (regulating surfactant protein production), and *CEBPD* (AT2 cell marker) (Fig. 3d). Loss of lineage identity during tumor development was shown in a recent study [47]. Thus, we used gene single sample set enrichment analysis (ssGSEA) to evaluate the activities of these two important pathways related to stem cell characteristics in GI and GII (Fig. 3f), and then performed GSEA analysis to verify the differential activation of stem cell related pathways between GI and GII (Additional file 1: Figure S8C and D). Compared with GI, the GII-upregulated genes were enriched in processes associated with rapid cell proliferation and a de-differentiated state (Additional file 1: Figure S8C and D). These results suggested that GI represented as more differentiated state while GII displayed a more stem cell-like phenotype. However due to the lack of temporal relationship between samples, we could not perform a trajectory analysis to check whether GI and GII corresponded to early and late-stage during tumor progression.

In order to investigate the epigenomic and transcriptomic changes in the continuum progression from differentiated state to de-differentiated state, we further divided GII tumor samples into GII.1 and GII.2 based on previous hierarchical clustering result and re-analyzed the epigenetic and transcriptomic changes among these three subgroups (Additional file 1: Figure S7E and F). GII.1 and GI had great differences in epigenome but not in transcriptome (Additional file 1: Figure S7G, third column), compared to the differential analysis between GII.2 and GI (Additional file 1: Figure S7G, first column). The epigenetic alterations in GII.1 compared to GI seemed like prior to the transcriptional changes between them during the progression. These results indicated that epigenetic regulators might be involved in the epigenome remodeling in GII.1 during the progression. Taken together, epigenetic heterogeneity led to the discovery of two LUAD subgroups and then different driver genes and biological pathways enriched in each LUAD subgroup, which can greatly help to explain the differences in clinical outcomes among patients.

### DEGs between GI and GII tumors can predict LUAD prognosis

To verify the utility of our LUAD classification model, we incorporated the transcriptomic data of 504 LUAD patients in TCGA database from diverse populations and tried to map each patient to GI or GII based on transcriptome similarity. We first calculated the ssGSEA scores of GI-specific and GII-specific genes in the RNA-seq profile of each TCGA-LUAD patient’ tumor sample, which were called the GI and GII scores, respectively. We designated the TCGA patients with high GI score and low GII score samples as GI-like, and the patients with low GI score and high GII score samples as GII-like, leaving the remainder as the intermediate group (intergroup). In total, we defined 143 GI-like, 142 GII-like, and 219 intergroup patients. Consistently, GI-like patients had significantly better OS than GII-like patients (Fig. 3g). We also noticed that the majority of GI-like patients were labeled as stage I patients by TCGA, while most GII-like patients were at later stages (II-IV, Fig. 3i). In particular, even for the stage I patients, the GII-like ones showed clearly worse prognosis compared to the GI-like ones (Fig. 3h). These findings indicated that our LUAD classification model could be potentially used as a reference to predict tumor progression and risk.

Additionally, genomic mutation analysis of TCGA-LUAD datasets revealed more gene mutations in GII-like patients than in GI-like patients (Fig. 3j). Especially, some tumor suppressor genes (TSGs) including *DLC1* and *SYNE1*, which have been found to be downregulated in our GII tumor samples compared to GI samples, were predominantly mutated in GII-like TCGA patients (Fig. 3j). This result indicated that loss of TSG function caused by gene mutation and downregulation can directly contribute to progression in tumorigenesis. Taken together, these results supported our new classification model and showed pronounced transcriptomic changes in these two epigenetic subgroups that correlate with the OS rate. The GI-GII score derivative from our classification model could be used to identify high-risk patients, especially in stage I, and, therefore, constituted an important supplement to current TNM staging system.

### Subgroup-specific core regulators determined transcription characteristics in GI and GII

**Fig. 4:**
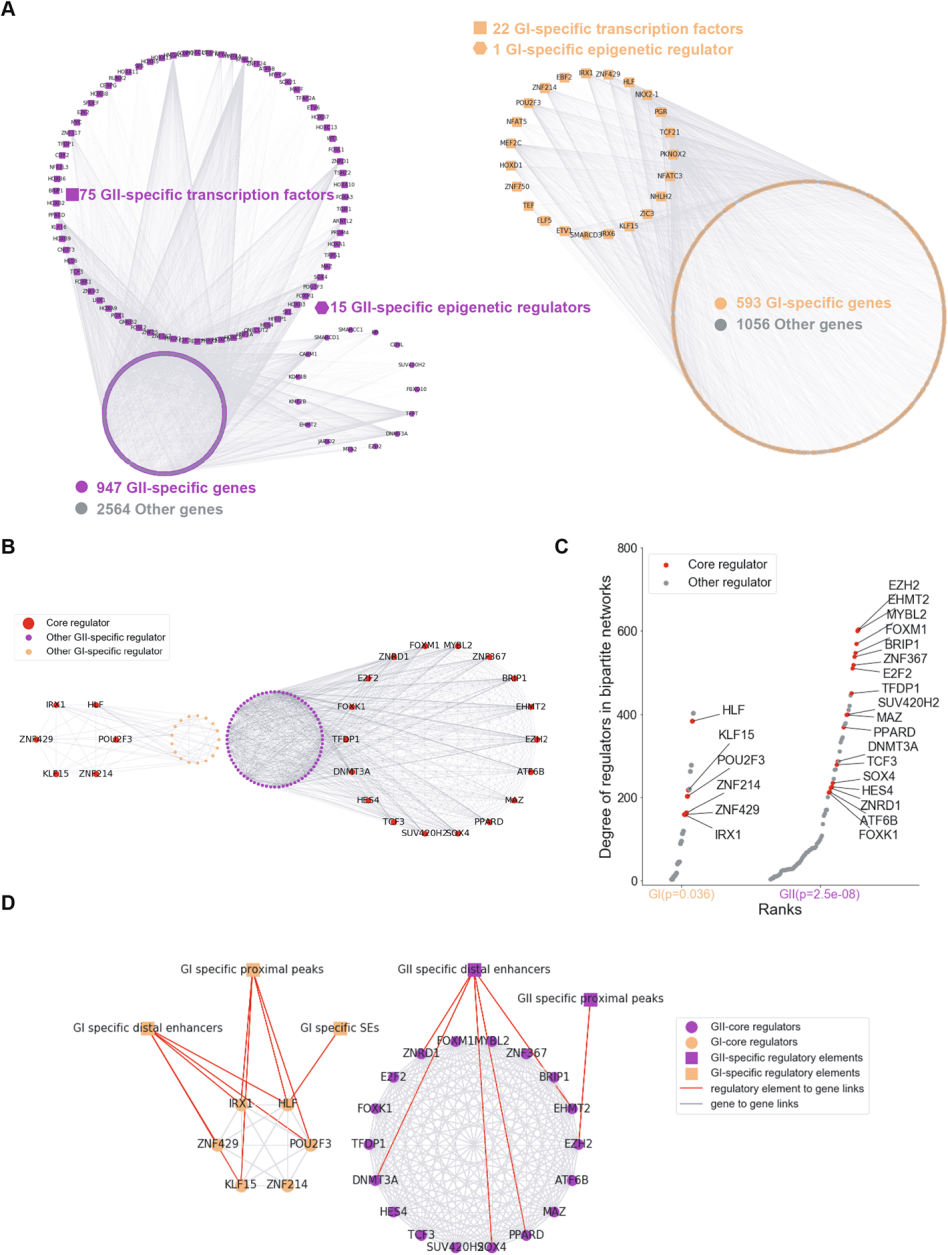
Co-expression networks for LUAD subgroups and group-specific core regulators. **a** Co-expression network (bipartite network) constructed in GI (orange) and GII (purple) based on gene expression correlations between differentially expressed transcription factors or epigenetic regulators and their target genes. **b** Co-expression network of differentially expressed regulators. Core regulators in GI (6 core regulators, left panel) and GII (18 core regulators, right panel), core regulators formed a highly connected clique in the regulator co-expression networks. **c** Core regulators(red) and other regulators(grey) with their degrees calculated from the bipartite networks in **a**, *p*-value determined by rank-sum test. **d** Upstream differential epigenetic elements participate in regulation in group-specific core regulator, including differential distal enhancers, differential proximal peaks, and differential super-enhancers

In order to explore key regulators driving the transcriptional differences between two subgroups, a co-expression network was constructed based on the differential expressed epigenetic regulators and transcription factors between GI and GII, which were considered regulators here, as well as their target genes identified by co-expression analysis. We found that the network formed two different sub-networks, which correspond to the two subgroups and were named as GI-specific network and GII-specific network, respectively (Fig. 4a). More importantly, GI-specific network contained 539 (50.4%) GI-specific genes, and GII-specific networks covered 947 (66.2%) GII-specific genes (Additional file 5: Table S4, Additional file 6: Table S5). These results indicated that the transcriptional characteristics of GI and GII were driven by different regulatory modules. We also found that the degrees of the transcription and epigenetic regulators showed a power law distribution (Additional file 1: Figure S9A), indicated that a small set of them had very high connectivity in the network, and thus might play important roles in driving the transcription characteristics of GI and GII. Then, we constructed the co-expression network of regulators and identified the core regulators of GI and GII, which not only had high connectivity with each other but also with other regulators (see methods). By this means, we identified 6 core regulators for GI and 18 core regulators for GII (Fig. 4b, c). In addition, 9 of the 18 GII-specific core regulators were annotated to cell cycle pathway (Additional file 1: Figure S9B), such as *MYBL2*, *FOXM1*, previously known to be related to cancer abnormal cell cycle [49]. In contrast, GI-specific core regulators were assigned to complement and coagulation cascades and metabolic related pathways (Additional file 1: Figure S9B), such as *POU2F3*, which has been identified as a good prognosis marker of LUAD. The better prognosis of high *POU2F3* expression patients was confirmed by The Human Protein Atlas [50], with a 46% 5-year survival rate compared to 34% 5-year survival rate of low *POU2F3* expression patients. The shift of core regulators might happen at the initial stage and drives the transition from GI to GII. We observed an upregulation of GII-specific core regulators *EZH2* and *EHMT2* in GII.1 compared to GI, and they were further upregulated in GII.2 (Additional file 1: Figure S9C). We also detected a progressive downregulation of GI-specific core regulators *HLF* and *IRX1* (Additional file 1: Figure S9C). Compared with GI samples, GII.1 upregulated genes were highly enriched in epigenetic regulation of gene expression, consistent with the function of GII-specific core regulators (Additional file 1: Figure S9D). Moreover, by integrating with our previously defined links from super-enhancers, distal enhancers and proximal H3K27ac peaks to gene s[51], a large fraction of the GI and GII-specific core regulators were found to be directly assigned to at least one differential epigenetic element (Fig. 4d). In summary, the transcriptional characteristics of the LUAD subgroups identified here were driven by a number of subgroup-specific core regulators, which potentially influenced the biological pathways related to clinical prognosis and contributed to LUAD progression. Meanwhile, subgroup-specific epigenetic elements acted as upstream factors driving the differential expression of these core regulators.

### Screening of genes regulated by GI-specific core regulators and in vivo validation reveals CLU as a potential tumor suppressor

**Fig. 5:**
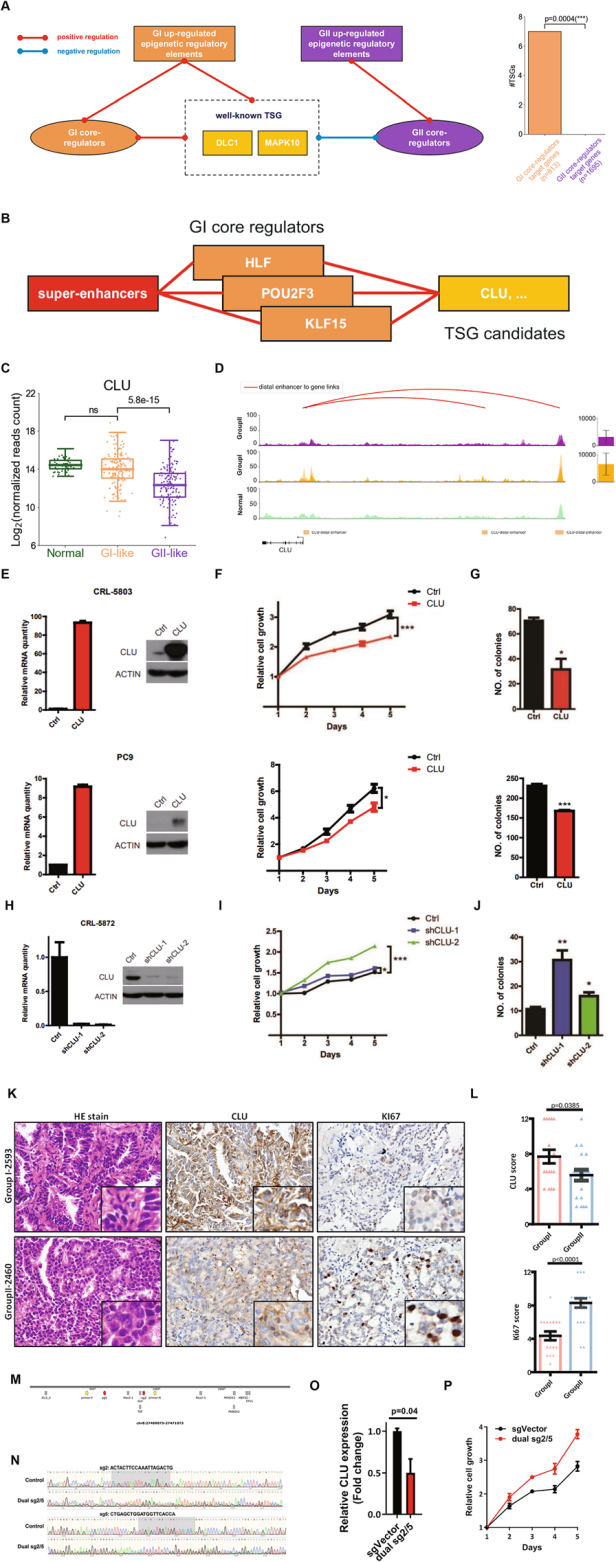
In vivo and in vitro validation of potential function of identified TSGs. **a** A schematic diagram illustrated how core regulators and epigenetic regulatory elements (including H3K27ac-marked distal enhancers, promoters, and super-enhancers) regulated well-known TSGs. Bar plot in the right indicated that there were more active TSGs in GI core regulator target genes than GII core regulator target genes. The *p*-value of Fisher-exact test showed in the plot. **b** The strategy used for selecting TSG candidates. Three super-enhancer-associated GI core regulators co-regulated genes were selected as TSG candidates. **c** Gene expression level of *CLU* in GI-like, GII-like, and normal samples in TCGA-LUAD cohort. The *p*-value of t-test showed in the plot. ns, not significant. **d** Track plots revealed *CLU* gene expression was regulated by GI upregulated distal enhancers. The *CLU* gene expression in GI and GII samples was shown on the right. **e** Real-time PCR quantification and Western blot detection of *CLU* in CRL-5803 (upper panel) and PC9 (lower panel) cells with or without *CLU* ectopic expression. **f** Cell proliferation assay in CRL-5803 (upper panel) and PC9 (lower panel) cells with or without *CLU* overexpression. **g** Soft agar colony formation assay in CRL-5803 (upper panel) and PC9 (lower panel) cells with or without *CLU* overexpression. **h** Real-time PCR quantification and Western blot detection of *CLU* in CRL-5872 cells with or without CLU knockdown. **i** Cell proliferation assay in CRL-5872 cells with or without *CLU* knockdown. **j** Soft agar colony formation assay in CRL-5872 cells with or without *CLU* knockdown. **k** Representative photos of HE and IHC staining of CLU and Ki-67 in GI and GII samples. **l** Statistical analyses of CLU and Ki-67 IHC scores in group I and group II samples. Data were shown as mean with S.E.M. Statistical analyses was calculated by two-tailed, unpaired *t*-test. **m** The TF binding motifs on the enhancer region. Grey boxes indicate TFs; red arrows, predicted sgRNAs; and yellow arrows, PCR primers for testing knockout efficiency. **n** Sequencing of the PCR products by reverse (R) primers to validate dual gRNA knockout efficiency. **o** *CLU* expression of the indicated sgRNAs by real-time PCR quantification. **p** Cell proliferation analysis of the indicated sgRNAs

After constructing a co-expression network dominated by the core regulators comprised of two subgroup-specific sub-networks, we asked whether the function of downstream genes regulated by these core regulators were directly linked with subgroup biological features. Making use of a previously compiled list of 49 TSGs whose expression was consistently downregulated in 11 different cancer types [52], we found that the GI core regulators target genes enriched more TSGs than GII core regulators target genes and the expression of these TSGs predominantly showed a positively correlation with GI-specific core regulators but a negatively correlation with GII-specific core regulators (Additional file 1: Figure S10A). Group-specific distal enhancers or SEs could control TSGs such as *DLC1* and *MAPK10* directly or indirectly via group-specific core regulators, which might help to explain the downregulation of these TSGs and tumor progression in GII (Fig. 5a). We then screened the genes directed linked with GI-specific core regulators to discover new candidate TSGs that can inhibit LUAD progression. As the top 3 GI core regulators were also regulated by SEs, we firstly searched the downstream genes co-regulated by *HLF*, *KLF15,* and *POU2F3* (Fig. 5b). A total of 38 protein-coding genes were identified (Additional file 7: Table S6). Interestingly, there were multiple well-known TSGs, including *MAPK10*, a well-studied TSG in esophageal cancer, cervical cancer, and breast cancer (Additional file 1: Figure S10B) [53–55]. To screen for progression associated TSGs, we further selected the candidate genes whose expression was only downregulated from GI-like to GII-like TCGA-LUAD tumor samples, but not from normal to GI-like ones. Three GI-specific genes were filtered out and we noticed *CLU*, one potential LUAD TSG which was included in our previous TSGs screening study [56]. During LUAD progression, *CLU* was significantly downregulated from GI-like to GII-like samples. However, there was no significant difference between normal and GI-like samples (Fig. 5c). The epigenetic analysis also showed that *CLU* was directly regulated by GI-specific enhancers (Fig. 5d). These results suggested that *CLU* might act as a TSG to inhibit LUAD progression. Our previous study showed that *CLU* knockout mediated by CRISPR/Cas9 technique in Kras^G12D^-based genetically engineered mouse model (GEMM) promoted LUAD malignant progression [56]. We further found that ectopic *CLU* expression in CRL-5803 and PC9 cells significantly inhibited cell proliferation and suppressed colony formation in soft agar (Fig. 5e–g). Conversely, *CLU* knockdown in CRL-5872 cells accelerated cell proliferation and promoted colony formation in soft agar (Fig. 5h–j). We also assessed the protein levels of CLU as well as Ki-67, a proliferation marker, in our cohort using IHC staining. Compared with GI tumors, the CLU level was significantly downregulated in GII tumors. In contrast, the Ki-67 level was conversely upregulated in GII tumors (Fig. 5k, l). We here identified *CLU* as a new TSG in LUAD which was related to tumor progression.

We then further investigated the regulatory relationship between *CLU* and associated enhancers. Using dual gRNA CRISPR system [57], we successfully performed knockout of a GI-specific enhancer, which contains the binding motif of GI core regulator *HLF*, in CRL-5872 cells (Fig. 5m, n). Consistent with our *CLU* knockdown results, *CLU* enhancer knockout significantly downregulated the expression of *CLU* and accelerated cell proliferation (Fig. 5o, p). Taken together, these data support that GI tumors display relatively high expression of TSGs, which may be directly regulated by GI-specific enhancers to suppress malignant progression and contribute to better patient prognosis.

## Discussion

In this study, we described the active H3K27ac landscape of 42 LUAD samples and paired normal lung tissues. Our data added new knowledge on pathogenic LUAD mechanisms at the epigenetic level and proposed a classification model based on intertumoral H3K27ac heterogeneity. Although genomic classification is mostly related to the current target therapy of LUAD, a majority of patients are lacking known therapeutic targets and need to be further characterized. The transcriptomic classification is also widely used. Various classification models in LUAD cohorts revealing morphologic features, gene expression signatures, and gene mutation landscapes in each subgroup were reported [15, 58, 59]. Although these subclassifications vary considerably from study to study, most of them can effectively predict patient prognosis and provide new insight on LUAD progression [60]. Recently, proteogenomics studies revealed LUAD molecular signatures and therapeutic vulnerabilities, which provided new insights of LUAD classification models [61, 62]. Other classification criterions like CpG island methylation were also investigated but still needed more comprehensive study [63].

Compared with previous methods, our H3K27ac-based classification provides new insights of epigenetic alterations in LUAD and investigates the transcriptomic features, co-expression network and core regulators which maintain the biological behavior in each subclass. Our epigenetic classification is highly associated with tumor histological features defined by previous reports and effectively predicts patient prognosis. Furthermore, our GI-GII score evaluating method can also effectively predict the prognosis of patients in the same TNM stage, thus could work as critical supplements to current TNM staging system in clinical application [7]. Plenty of biomarkers of LUAD have been found by our and others’ previous studies including many oncogenes, but few of them are valuable therapeutic targets as most biomarkers lacking effective and specific inhibitors, thus hindering the application of these findings. The relevant epigenetic elements and upstream core regulators, which are identified in our study, seem to be alternative targets to inhibit the expression of oncogenes. *BRD4* and *CDK7* are key factors for the function of epigenetic elements such as enhancers, and their inhibitors, JQ1 and THZ1, globally inhibit the expression of associated genes and suppressed lung cancer cell proliferation [32, 64]. Recently, the FDA has approved tazemetostat, the first inhibitor of *EZH2* (a top core regulator in GII), for treating epithelioid sarcoma, indicating *EZH2* as a potential target in LUAD [65].

In our study, a transformation process might occur from GI to GII during tumor progression, including the loss of expression of the LUAD differentiation marker and gradually inactivation of TSGs. This potential transformation process might explain the invasive behavior and worse prognosis in GII. Similar transformation process has been reported by Lindsay et al. in mouse model [47], but the transformation process from GI to GII seems very complicated. The identification and intervention of early changes are important because the prognosis of lung cancer is completely different between early-stage and late-stage patients [66]. The existence of a GII.1 subgroup hints that epigenetic alterations might act as pioneers in lung cancer progression, consistent with a recent study [47]. However, further studies by more extensive investigations in larger cohorts, direct tracer study, and pioneer gene screening are necessary to confirm this hypothesis.

In this study, we have identified *CLU* as a potential LUAD TSG. Previous study has proposed controversial functions of *CLU* in tumorigenesis, e.g., *CLU* works as a proto-oncogene in prostate cancer and colorectal cancers whereas as a TSG in lung cancer [67–71]. In a functional screening of the TSGs using CRISPR/Cas9 knockout platform in *Kras*^G12D^-based GEMM, we have previously identified *CLU* as an important tumor suppressor. Interestingly, we here find that *CLU* is simultaneously regulated by 3 top SE-associated GI core regulators, highlighting the potential function of *CLU* in LUAD malignant progression. Our in vitro study further supports the tumor-suppressive function of *CLU*. Future efforts will be interesting to look into in-depth link between *CLU* and SE-associated core regulators.

There were some limitations of this study. Previous studies reported several forms of histone modification, mainly involving the methylation or acetylation on K4, K9, K27, K36, and K79 on histone3 [24]. Due to the limitation of tissue samples, our study only focuses on H3K27ac, a hallmark of active of enhancer and promoter. Of course, other histone modifications such as H3K27me3 level will be interesting to explore in future in context of H3K27ac dynamic changes. We have used the transcriptomic profiles of a total 504 TCGA-LUAD patients without the H3K27ac profiling to validate our classification. More comprehensive study involving multiple histone modifications like H3K27me3 will be interesting to investigate in larger cohorts. Moreover, the traditional method of ChIP-seq to detect epigenetic changes requires high quality and quantity of tumor samples. However, the amount of available tissue is highly restricted especially in early-stage tumors and biopsy samples, which is also a main restriction to cohort size in such studies [72]. Future efforts will be interesting to take advantage of some newly developed techniques including CUT&Tag which requires only a small amount of samples or even single cells [73].

## Conclusions

We here describe the epigenetic alterations during LUAD tumorigenesis and provide a new classification model to predict patients’ prognosis. Through comprehensively analyses of epigenomic and transcriptomic features, we have constructed the co-expression networks that determine subgroup-specific biological characteristics. We also reveal epigenetic modifications, especially super-enhancers, and core regulators in regulating tumor progression, which potentially serve as novel therapeutic targets of LUAD. Loss of function of various TSGs is prominent in GII group, which might promote the LUAD progression. Our data further identify *CLU* as an important TSG in inhibiting LUAD progression.

## Methods

### Sample collection

This study was approved by the Ethics Committee of Fudan University Shanghai Cancer Center. A consent form was signed by every patient who received surgical resection for LUAD or by his/her legal representative before surgery. The tumor samples and adjacent normal samples (> 2 cm from the tumor) were collected immediately after resection of the tumor and stored in liquid nitrogen before subsequent analyses. Tumor tissues were subjected to a pathological review to confirm the histology and the tumor cell content of each tumor tissue. Forty-two patients were included in this study.

### ChIP-seq in tissues

H3K27ac ChIP-seq was performed using an anti-H3K27ac antibody (abcam, ab4729). In brief, frozen LUAD and normal tissues were submersed in RPMI-1640 medium (Corning, 10-040-CVR), cut into small pieces, and homogenized on ice with a Dounce homogenizer. The tissue homogenate was filtered with a 70-μm cell strainer and fixed in 1% formaldehyde at room temperature for 10 min. The tissue pellet was collected through centrifugation, washed twice with cold PBS, and then incubated with lysis buffer on ice for 20 min. The tissue lysate was sonicated, and DNA was sheared to an average size of 100–300 bp. Approximately 10% of the total tissue lysate was collected as input, while the other portion was first precleared with uncoupled Protein-A Dynabeads (Novex, 10002D) and incubated with H3K27ac-coupled Protein-A Dynabeads for 6 h in a cold room. Then, the Dynabeads were collected using a magnet track, and chromatin was eluted. The immunoprecipitant (IP) and input chromatin were incubated at 65 °C overnight and treated with RNase A and proteinase K, and then the DNA was purified (Tiangen, DP214-03). The DNA was then processed for NGS library construction and sequenced.

### Peak calling and classification of LUAD patients

H3K27ac ChIP-seq reads were mapped to the human reference genome hg19/GRCh37 using Bowtie v1.2.2. Only uniquely mapped reads were retained. Duplicated reads were removed before downstream analysis. H3K27ac peaks were called by using MACS v1.3.7, and the 30,000 most significant peaks associated with each sample were retained. All H3K27ac peaks were merged into a consensus list of peaks, and reads finally within these peaks were counted by using MAnorm2-utils [29]. Differential H3K27ac sites between tumor and normal samples (or between GII and GI) were identified using MAnorm2 [29]. Those sites with adjusted *p*-value below 0.05 and fold change greater than 2 were defined as differential ones.

To perform a classification of tumor ChIP-seq samples, we first identified peaks that were associated with hyper-variable signals across all the tumor samples by using MAnorm2 (*p*-value ≤ 0.01) [29]. These peaks were defined as tumor hyper-variable peaks (HVPs). Peaks in sex chromosomes were removed for downstream analysis, and principle component analysis (PCA) was performed on the tumor HVPs. Permutation analysis was used to determine significant principal components (PCs). Among the significant PCs, which were the first 5 PCs, only PC1 showed significant association with clinical information of patients (e.g., ANOVA test for lymph node invasion gave a *p*-value of 0.04). We therefore used only PC1 for hierarchically clustering the tumor samples. ConsensusClusterPlus algorithm was used to determine the optimal number of clusters [74], and we observed that k = 2 led to clearly more stable clustering results compared to k = 3 (Additional file 1: Figure S5A). Considering the limited cohort size, we have chosen k = 2 and have classified the tumor samples into group I (GI) and group II (GII).

### Peak saturation analysis

To better understand whether our epigenetic profiling adequately captured the epigenome landscape of paired primary LUADs and normal samples, we used the peaks of interest (not in the black list from https://www.encodeproject.org/files/ENCFF001TDO/) in each sample to calculate the number of discrete peaks and the number of novel peaks gained with increasing sample number. This was performed across 100 permutations of the tumor and normal samples (Additional file 1: Figure S1A).

### Super-enhancer analysis

ROSE was employed to identify super-enhancers (SEs) in each sample. A 12.5-kb stitching distance was used to connect the proximal cluster of H3K27ac peaks into continuous enhancer regions, and regions around the TSS within 2.5 kb were excluded. We found that tumor-specific peaks and normal-specific peaks were mutually exclusive, and with increasing SE size (the length of SE), the fraction of differential H3K27ac sites was reduced. Thus, we used Fisher’s exact test to define differential SEs based on the relative enrichment of differential H3K27ac sites compared to the background (Additional file 1: Figure S2). SEs were mapped to genes based on the correlation between the SE score and H3K27ac signal intensities of the promoter (peaks within 2.5 kb of the TSS) or the gene expression level within ± 500 kb using a adjusted *p*-value of 0.01 and 0.05, respectively. Any SE without a linked gene was linked to the most highly correlated or closest gene. For each SE, SE score is the average normalized signal intensities of enhancers within this SE.

### Identification of distal enhancer-linked genes

To assign distal enhancers to genes, we used a correlation-based method. All possible interactions between the distal enhancers and TSSs within 500 kb were identified. For each interaction, we calculated the Pearson’s correlation coefficient (PCC) between H3K27ac signal intensities and gene expression levels (Additional file 1: Figure S6A). And we constructed a null distribution of non-specific correlations using random pairs of distal enhancers and genes (not within ± 500kb or not in the same chromosome) to determine the significance of PCC (Additional file 1: Figure S6B). Then, we calculated the mean and standard deviation of these non-specific correlations. We used a normal distribution with the previously calculated mean and standard deviation to compute the *p*-value for each correlation and adjusted for multiple hypotheses using the Benjamini-Hochberg procedure (false discovery rate, FDR). Then, all correlations with an FDR below 0.05 were defined as distal enhancers linked genes.

### Pathway enrichment analysis

To identify the epigenetic signatures, GSEA was performed based on the PCC of PC1 and gene expression using the gseapy Python package, which implements GSEA on a preranked gene list, and the Molecular Signatures Database (MsigDB). For the functional characterization of differential enhancers-associated genes, DEGs, and differential SE-associated genes, we used DAVID [75] and Metascape [76] to identify pathways that were significantly represented in the gene list from our dataset.

### RNA-seq and data analysis

Tissue samples were homogenated in lysis buffer and RNA was extracted as user manual (AllPrep DNA/RNA Mini Kit, Cat.no 80204, Qiagen). RNA samples were then processed to library construction. RNA and library DNA quantity were evaluated with Qubit 3.0 and suitable kit. RNA and library size distributions were measured with Aglient Bioanalyzer 2100 or QIAxcel system. High-throughput sequencing was performed with Illumina Hi-seq X10 system. RNA-seq reads were aligned to hg19 human genome and transcript annotation (GENCODE). Gene raw read counts were calculated via feature counts in R package Subread. Differentially expressed genes (DEGs) analysis was perform using DESeq2 [77]. DESeq2 was run with the formula “~ group + gender”, where group has levels “Group I” and “Group II” and “gender” has levels “male” and “female”. Genes with adjusted *p*-value less than 0.05 defined as DEGs.

### TCGA sample classification based on RNA-seq

GI- and GII-specific genes were identified using DEseq2, and ssGSEA was used to calculate the sample wise gene set enrichment score of GI- and GII-specific genes in each TCGA sample (called the GI score and GII score, respectively). We used 1/3 and 2/3 quantiles of the GI score and GII score across all samples to divide the TCGA samples into three groups, high GI score and low GII score samples as GI-like group, low GI score and high GII score samples as GII-like group, and the remainder as the intergroup. Then, the R package survminer was used to perform a survival analysis of these groups. Gene expression matrix of TCGA-LUAD was downloaded via TCGA-biolinks [78].

### Co-expression networks construction

We identified transcription factors(TFs) and epigenetic regulators based on functional annotation databases [74, 79] and selected those were DEGs as regulators, we used these regulators as key nodes to expand the networks based on significant positive correlation in gene expression (FDR ≤ 0.01) of these regulators and other genes. Co-expression network visualized using Python package networkx. To identify core regulators among these differentially expressed regulators, which not only had high connectivity with each other but also with other regulators, the co-expression network of differentially expressed regulators was constructed based on significant gene expression correlation (FDR ≤ 0.01). Core regulator clique was defined as regulator clique with the highest clique score in the regulators co-expression network and regulators in this clique were defined as core regulators. Two important definitions: “regulator score” was defined as the number of cliques that in which regulator participated. “clique score” for a clique was defined as the sum of “regulator score” of all regulators in that clique. We first calculated the “regulator score” of each regulator in the regulators co-expression network, then we calculated the “clique score” of each clique in this network, finally selected regulators from the clique with highest “clique score” as core regulators.

### Cell culture and functional assay

The HEK-293T cell and human NSCLC cell lines including PC9, CRL-5803, and CRL-5872 were purchased from the American Type Culture Collection and cultured in DMEM supplemented with 8% FBS. All cell lines used in this study were mycoplasma-free. The HEK-293T cells were used for lentivirus production for ectopic *CLU* expression and *CLU* knockdown as previously described [56]. MTT assay was performed following the manufacture manual. Briefly, 2000 cells were seeded on 96-well plates, and the viability of cells was measured daily for 5 days. All experiments were performed in triplicate. The CRL-5803 and PC9 cells were virally infected for ectopic *CLU* expression and the CRL-5872 for sh*CLU* knockdown. These cell lines together with the parental cells were then used for MTT assay and the colony formation in soft agar. For soft agar assay, 5000 cells were mixed with 0.2% agar containing growth medium and layered onto 1% agar containing growth medium in 6-well plates. Medium were changed every 3 days and the colonies were stained with 0.005% crystal violet and counted in 3 weeks.

### Plasmid construction

For gene expression in cell lines, the coding sequences of CLU were PCR amplified and cloned into pCDH-puro vector. For dual gRNA CRISPR screening, we insert another U6-filler-TA into pSECC, thus two gRNAs can be cloned into one vector. Briefly, the first gRNA, sg2, was cloned into BsmBI sites of pSECC vector. For the second gRNA, sg5, the U6-filler-TA sequence was amplified from pSECC and inserted into the vector pCDNA3.1 between EcoRI and XhoI which we then called pCDNA3.1-linker. The sg5 was cloned into BsmBI sites of pCDNA3.1-linker and then the U6-sg5-TA was cloned into pSECC-sg2 using ECoRI and XhoI. All primers used are listed as follows: For q-RT PCR analysis: qF-CLU: CCAATCAGGGAAGTAAGTACGTC, qR-CLU: CTTGCGCTCTTCGTTTGTTTT. For PCR sequencing: primer-F: CCATTCAGAACTAGGTTCTGACC, primer-R: GTGGCCTCTGTGTGCTTGTCT. Dual sgRNA target sites: sg2: ACTACTTCCAAATTAGACTG, sg5: CTGAGCTGGATGGTTCACCA. ShRNA target sites: shCLU-1: AACCAGAGCTCGCCCTTCTAC, shCLU-2: AGCAGCTGAACGAGCAGTTTA.

### Quantitative real-time PCR

Total RNA was extracted using TRIzol reagent (Invitrogen) and retrotranscribed into first strand cDNA using PrimeScript RT reagent Kit with gDNA Eraser (TaKaRa). The cDNAs were subjected to real-time PCR with gene-specific primers using SYBR-Green Master PCR mix (Roche). Actin was served as internal controls.

### Immunohistochemistry (IHC)

IHC was performed as described previously [80]. Paraffin-embedded clinical tissues were incubated with the following antibodies: anti-SOX9 (abcam, ab185966; 1:1000 dilution), anti-RUNX2 (abcam, ab192256; 1:1000 dilution), anti-TTF1 (abcam, ab76013; 1:250 dilution), anti-Ki67 (Proteintech, 27309-1-AP; 1:5000 dilution), anti-SOX4 (abcam, ab243041, 1:1000 dilution), anti-RUNX1 (abcam, ab240639, 1:2000 dilution), anti-SIX1 (abcam, ab252224, a:100 dilution), and anti-Clusterin (abcam, ab92548; 1:200 dilution). The immunostaining was reviewed and scored blindly. The scoring system for grading expression level was reported previously [81]. The score of each sample was multiplied by the grading of intensity and staining area.

## Supplementary Information

**Additional file 1: Supplementary figures.** All figures explain the content of this study but were not listed as figures in the article.

**Additional file 2: Table S1.** Sample clinical information.

**Additional file 3: Table S2.** Normal tissue specific SEs and associated genes.

**Additional file 4: Table S3.** Tumor tissue specific SEs and associated genes.

**Additional file 5: Table S4.** The co-expression network constructed based on the GI specific regulators.

**Additional file 6: Table S5.** The co-expression network constructed based on the GII specific regulators.

**Additional file 7: Table S6.** 38 protein-coding genes regulated by top 3 GI regulators.

**Additional file 8: Table S7.** Hyper variable peaks identified in tumor samples.

**Additional file 9: Table S8.** Hyper variable peaks identified in normal samples.

**Additional file 10: Table S9.** Enrichment results of normal specific SEs associated genes by metascape.

**Additional file 11: Table S10.** Enrichment results of tumor specific SEs associated genes by metascape.

**Additional file 12.** Review history.

## Data Availability

All the raw sequencing data of ChIP-seq and RNA-seq generated in this study have been deposited into the European Genome-phenome Archive (EGAD00001007066, https://ega-archive.org/datasets/EGAD00001007066) [82]. The transcriptomic data, survival data, and genomic mutation data of TCGA-LUAD here is in whole or part based upon data generated by the TCGA Research Network: https://www.cancer.gov/tcga.
