# Supplementary material for: A systematic dissection of the epigenomic heterogeneity of lung adenocarcinoma reveals two different subclasses with distinct prognosis and core regulatory networks
Source: Genome Biol. 2021 May 17;22:156. doi: 10.1186/s13059-021-02376-1 (PMC8127276; doi:10.1186/s13059-021-02376-1)
Supplement: Supplementary file 1 — Additional file 1: Supplementary figures. All figures explain the content of this study but were not listed as figures in the article. [file 13059_2021_2376_MOESM1_ESM.pdf]

Fig. S1

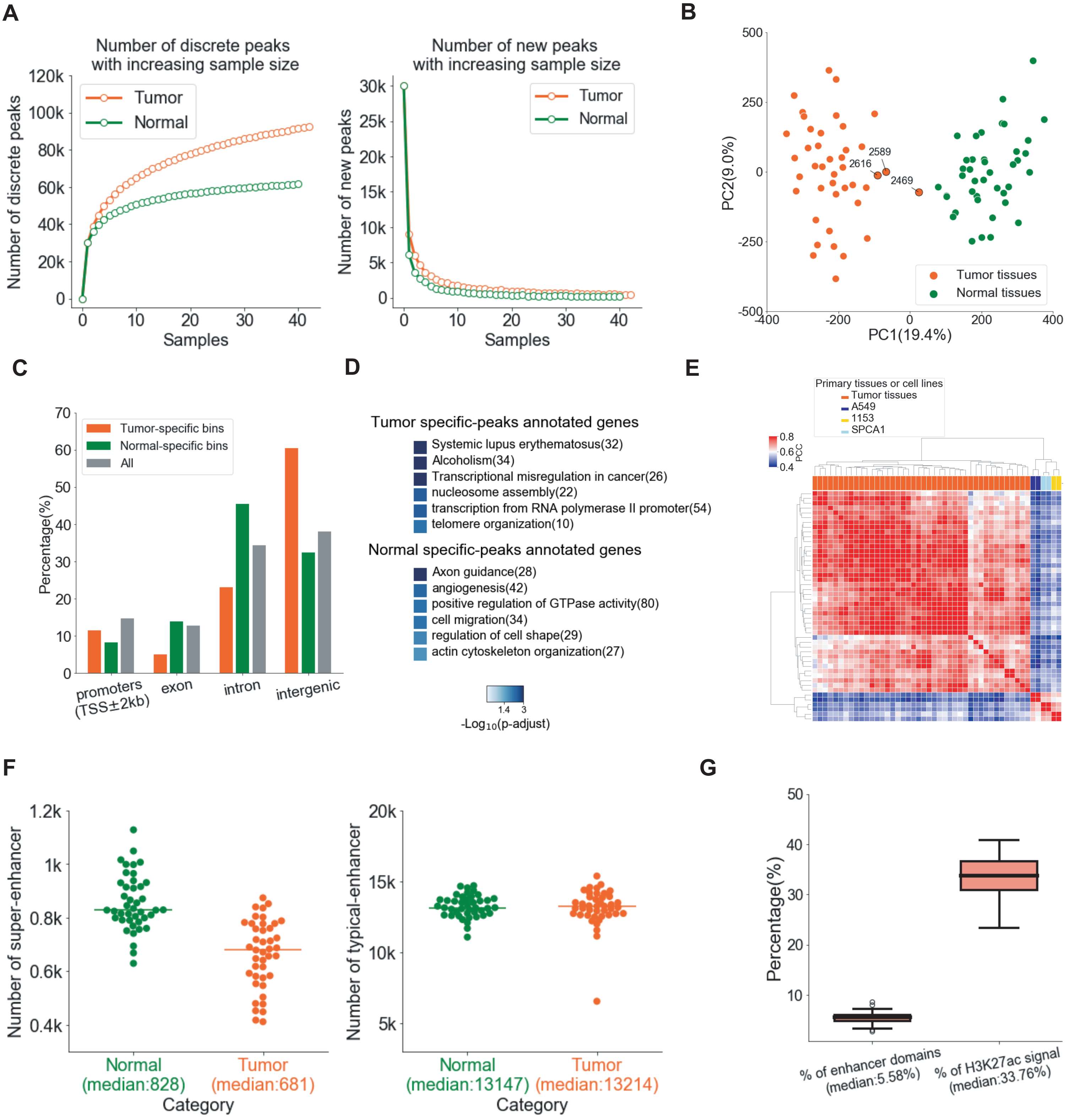
A
Number of discrete peaks with increasing sample size
Number of discrete peaks
Tumor
Normal
Samples
Number of new peaks with increasing sample size
Number of new peaks
B
PC2(9.0%)
PC1(19.4%)
2589
2616
2469
Tumor tissues
Normal tissues
C
Tumor-specific bins
Normal-specific bins
All
Percentage(%)
promoters (TSS±2kb)
exon
intron
intergenic
D
Tumor specific-peaks annotated genes
Systemic lupus erythematosus(32)
Alcoholism(34)
Transcriptional misregulation in cancer(26)
nucleosome assembly(22)
transcription from RNA polymerase II promoter(54)
telomere organization(10)
Normal specific-peaks annotated genes
Axon guidance(28)
angiogenesis(42)
positive regulation of GTPase activity(80)
cell migration(34)
regulation of cell shape(29)
actin cytoskeleton organization(27)
-Log10(p-adjust)
E
Primary tissues or cell lines
Tumor tissues
A549
1153
SPCA1
PCC
F
Number of super-enhancer
Normal (median:828)
Tumor (median:681)
Category
Number of typical-enhancer
Normal (median:13147)
Tumor (median:13214)
Category
G
Percentage(%)
% of enhancer domains (median:5.58%)
% of H3K27ac signal (median:33.76%)

Fig. S2

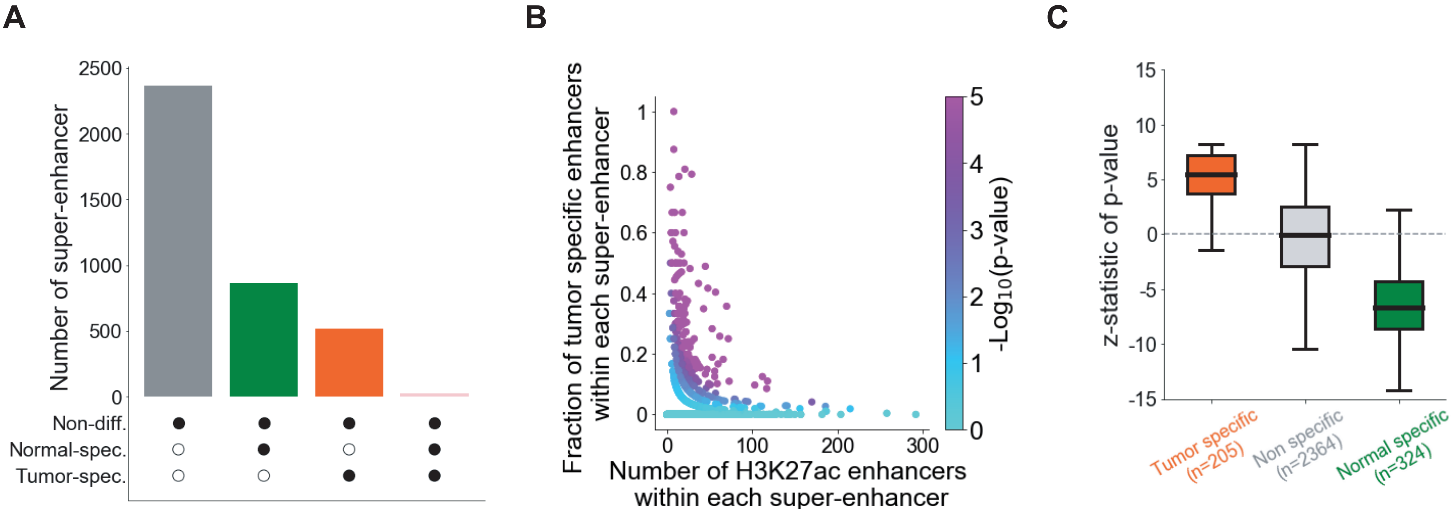

D

	super-enhancer	typical-enhancer	sum
differential sites	5,814	5,760	11,574
non-differential sites	90,906	138,734	229,640
Odd-ratio	1.54		
p-value	7.2E-113		

Fig. S3

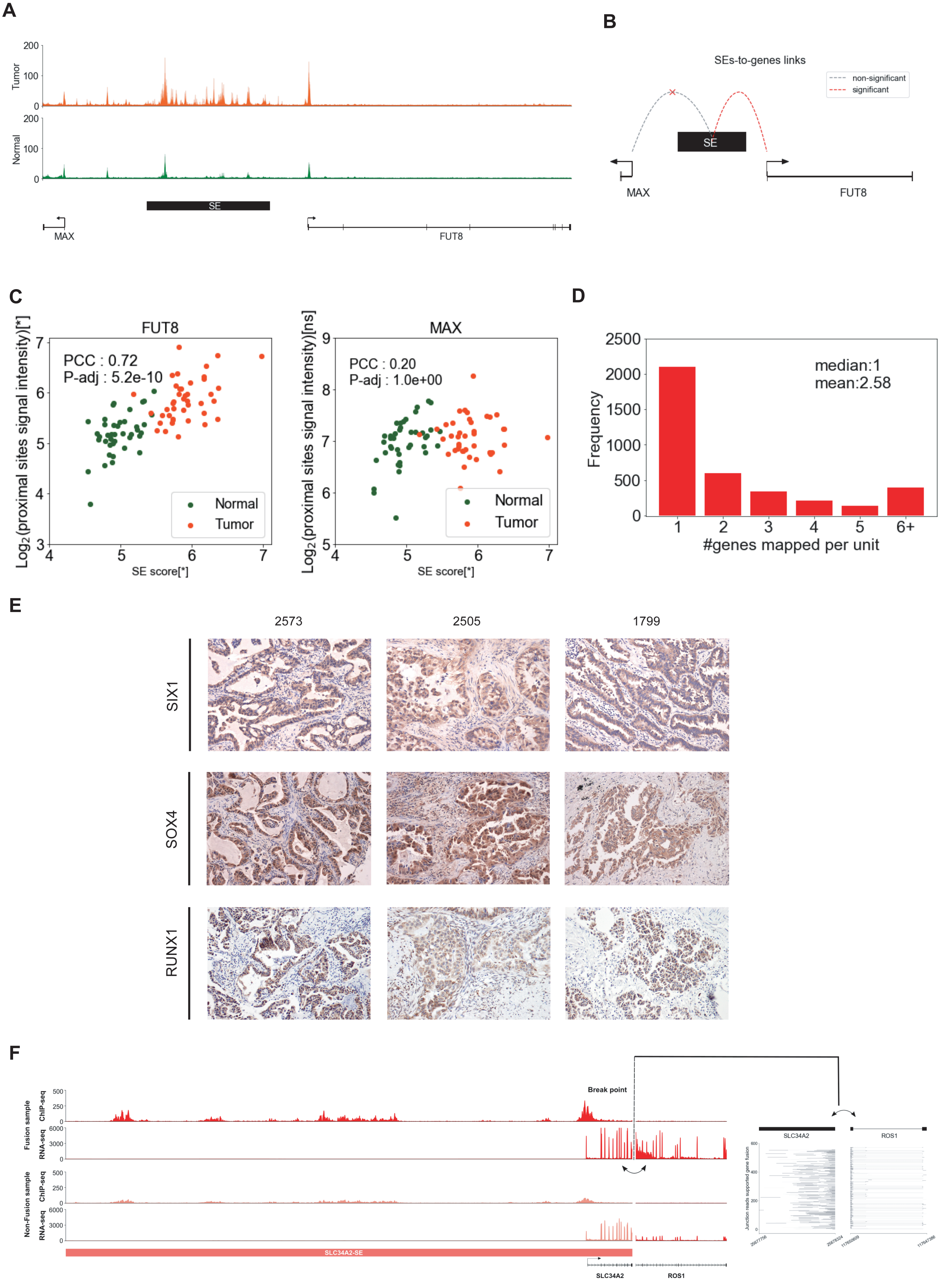
A
Tumor
Normal
200
100
0
SE
MAX
FUT8
B
SEs-to-genes links
non-significant
significant
SE
MAX
FUT8
C
FUT8
PCC : 0.72
P-adj : 5.2e-10
Normal
Tumor
Log2(proximal sites signal intensity)[*]
SE score[*]
MAX
PCC : 0.20
P-adj : 1.0e+00
Normal
Tumor
Log2(proximal sites signal intensity)[ns]
SE score[*]
D
median:1
mean:2.58
Frequency
#genes mapped per unit
1
2
3
4
5
6+
E
2573
2505
1799
SIX1
SOX4
RUNX1
F
Break point
Fusion sample
ChIP-seq
RNA-seq
Non-Fusion sample
ChIP-seq
RNA-seq
SLC34A2-SE
SLC34A2
ROS1
Junction reads supported gene fusion

Fig. S4

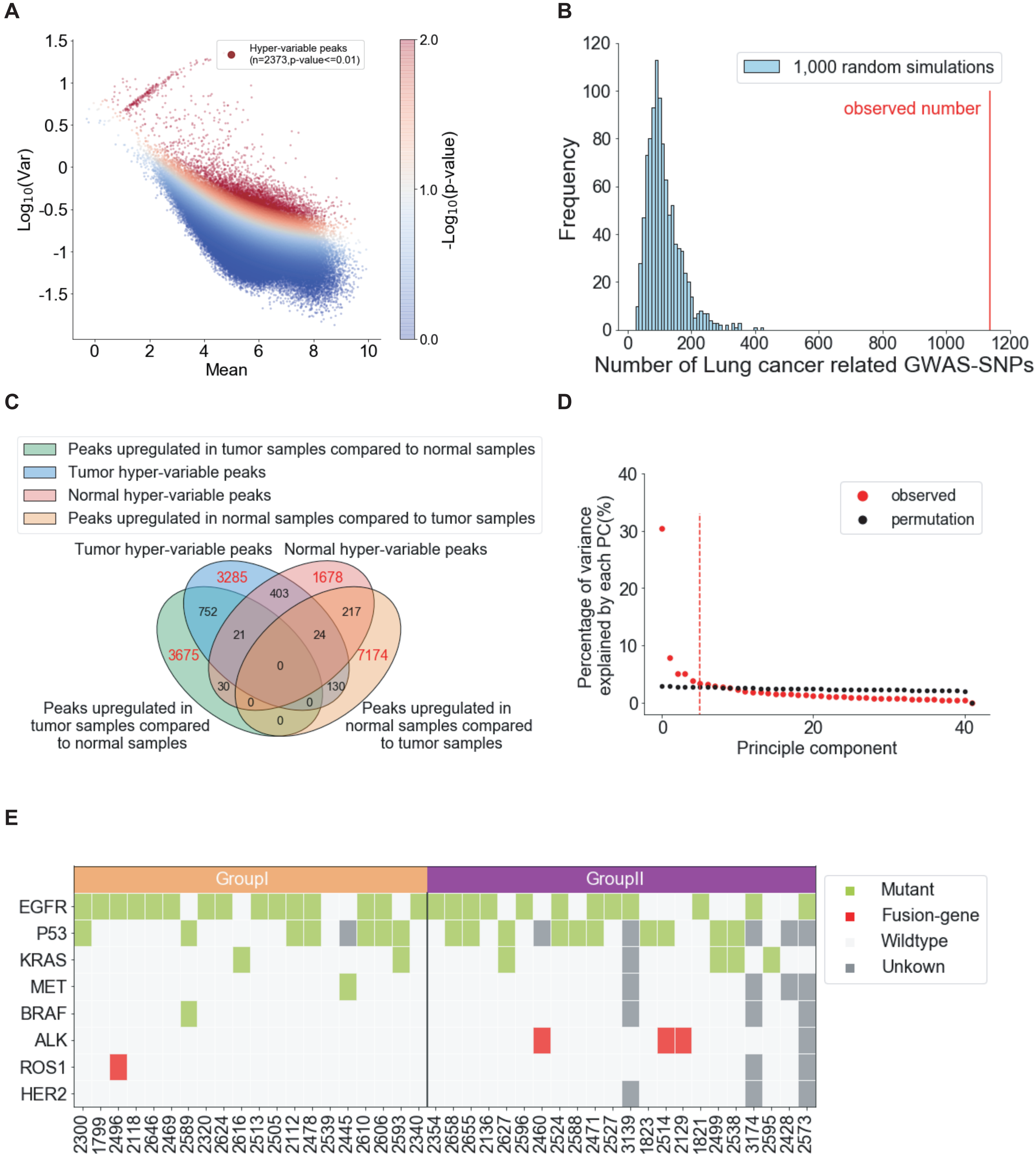
A
Hyper-variable peaks (n=2373,p-value<=0.01)
Log10(Var)
Mean
-Log10(p-value)
B
1,000 random simulations
observed number
Frequency
Number of Lung cancer related GWAS-SNPs
C
Peaks upregulated in tumor samples compared to normal samples
Tumor hyper-variable peaks
Normal hyper-variable peaks
Peaks upregulated in normal samples compared to tumor samples
Peaks upregulated in tumor samples compared to normal samples
Peaks upregulated in normal samples compared to tumor samples
3285
1678
403
752
217
21
24
3675
7174
0
30
130
0
0
0
D
observed
permutation
Percentage of variance explained by each PC(%)
Principle component
E
GroupI
GroupII
EGFR
P53
KRAS
MET
BRAF
ALK
ROS1
HER2
Mutant
Fusion-gene
Wildtype
Unkown
2300 1799 2496 2118 2646 2469 2589 2320 2624 2616 2513 2505 2112 2478 2539 2445 2610 2606 2593 2340 2354 2658 2655 2136 2627 2596 2460 2524 2588 2471 2527 3139 1823 2514 2129 1821 2499 2538 3174 2595 2428 2573

Fig. S5

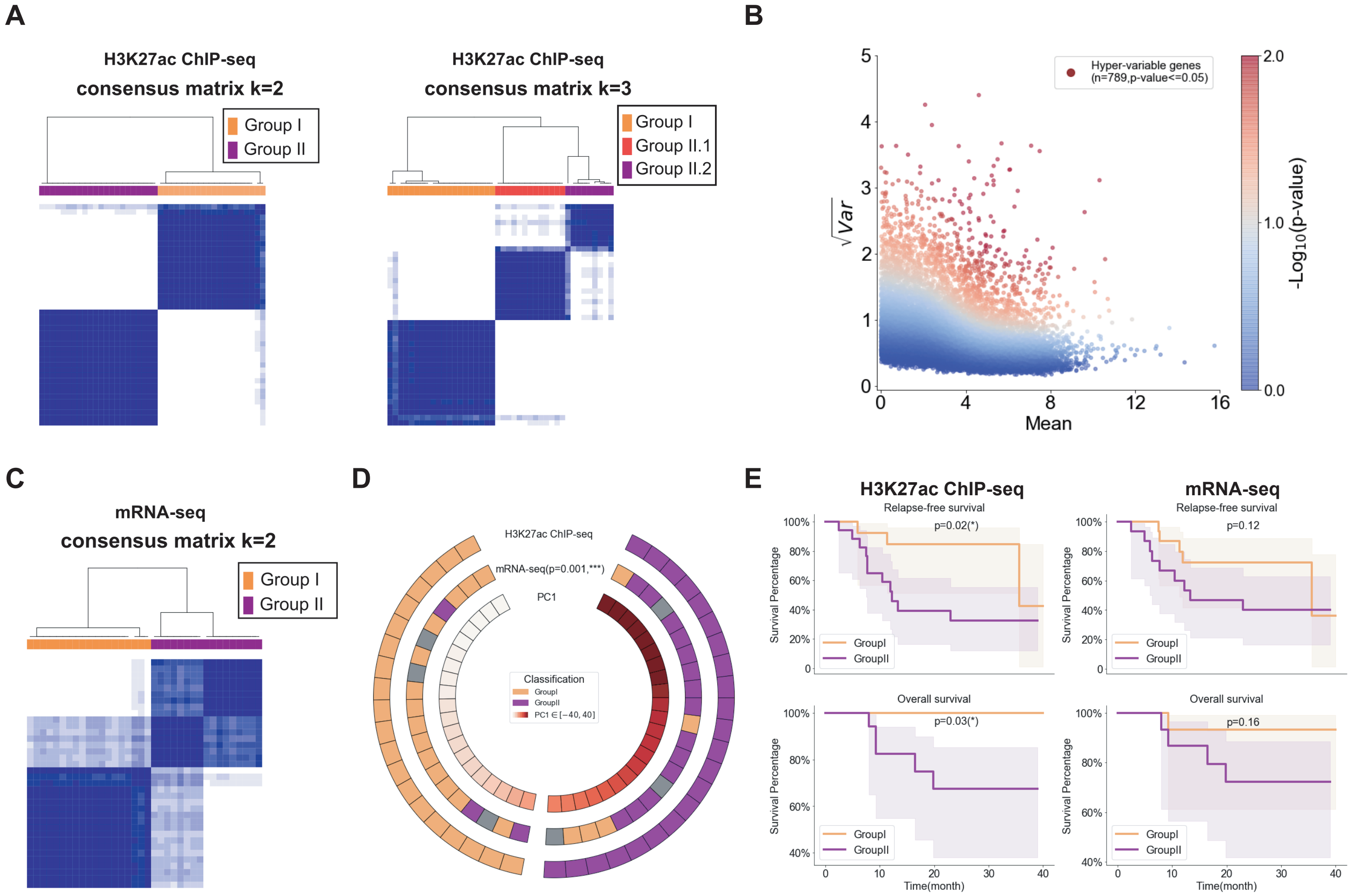
A
H3K27ac ChIP-seq
consensus matrix k=2
Group I
Group II
H3K27ac ChIP-seq
consensus matrix k=3
Group I
Group II.1
Group II.2
B
Hyper-variable genes
(n=789,p-value<=0.05)
√Var
Mean
-Log10(p-value)
C
mRNA-seq
consensus matrix k=2
Group I
Group II
D
H3K27ac ChIP-seq
mRNA-seq(p=0.001,***)
PC1
Classification
GroupI
GroupII
PC1 ∈ [−40, 40]
E
H3K27ac ChIP-seq
Relapse-free survival
p=0.02(*)
Overall survival
p=0.03(*)
mRNA-seq
Relapse-free survival
p=0.12
Overall survival
p=0.16
Survival Percentage
GroupI
GroupII
Time(month)

Fig. S6

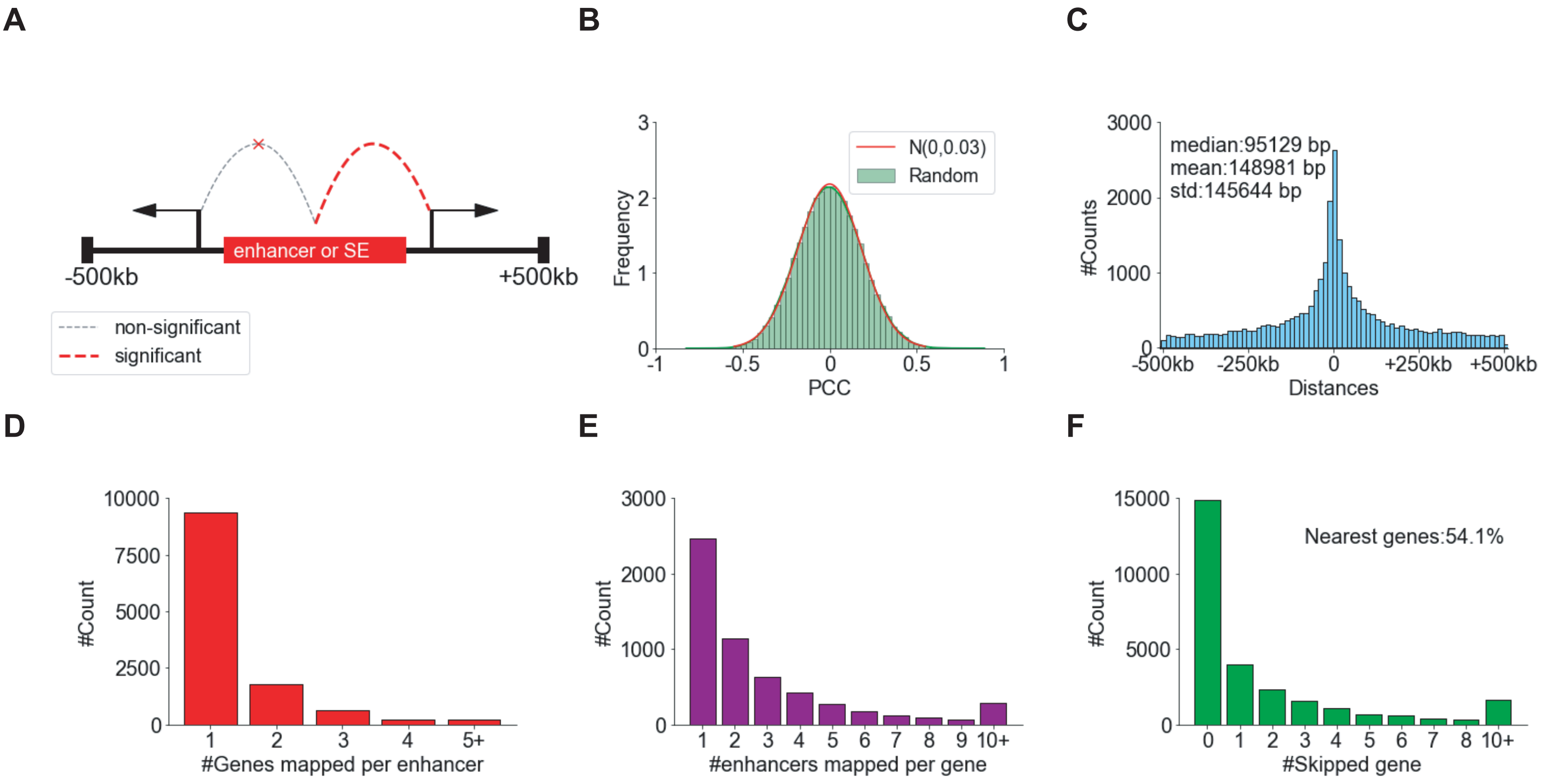
A
B
C
enhancer or SE
-500kb
+500kb
non-significant
significant
N(0,0.03)
Random
Frequency
PCC
median:95129 bp
mean:148981 bp
std:145644 bp
#Counts
Distances
-500kb
-250kb
+250kb
+500kb
D
E
F
#Count
#Genes mapped per enhancer
#enhancers mapped per gene
Nearest genes:54.1%
#Skipped gene

Fig. S7

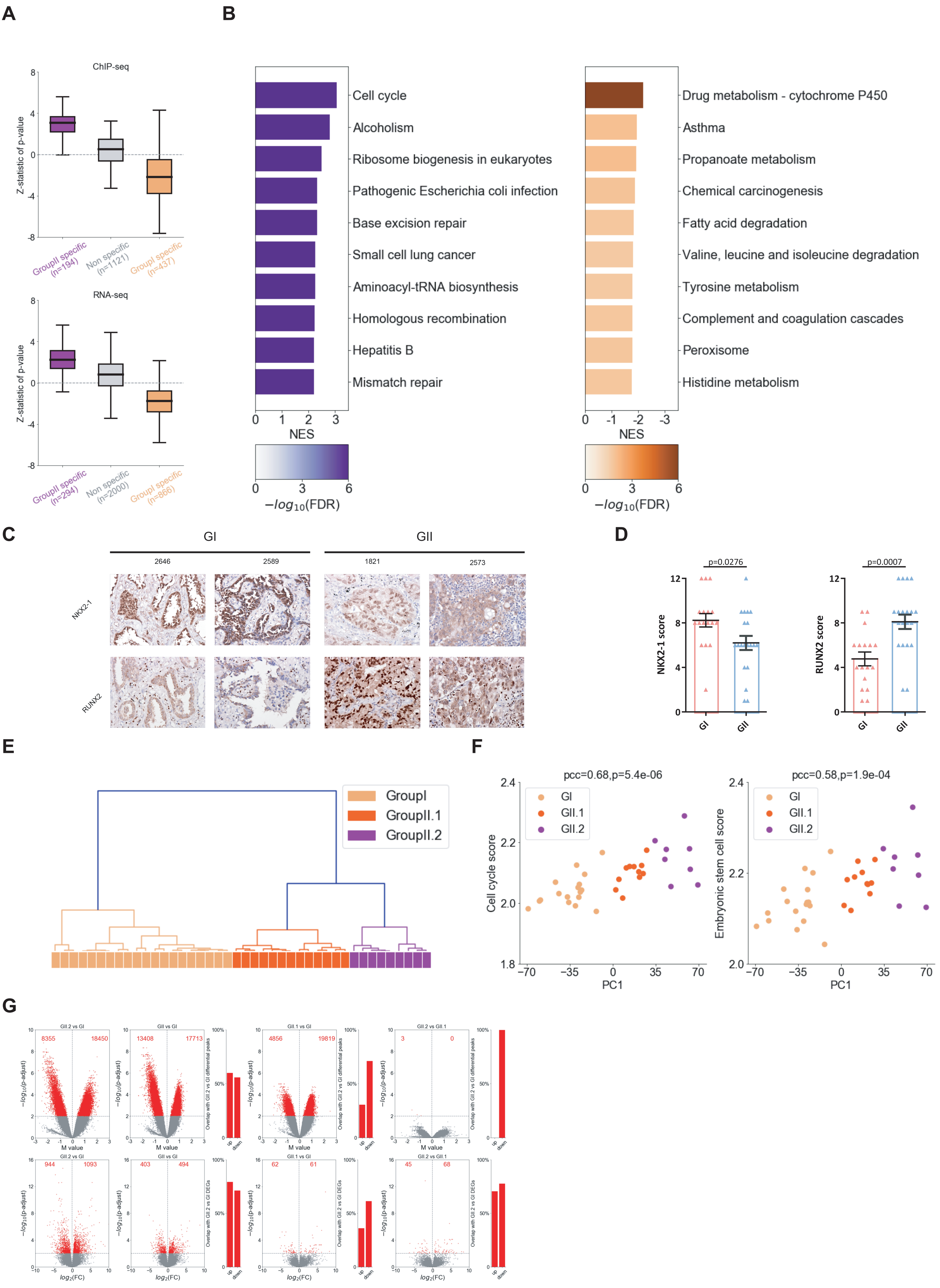

A
B
ChIP-seq
RNA-seq
Z-statistic of p-value
GroupII specific (n=194)
Non specific (n=1121)
GroupI specific (n=437)
GroupII specific (n=294)
Non specific (n=2000)
GroupI specific (n=866)
Cell cycle
Alcoholism
Ribosome biogenesis in eukaryotes
Pathogenic Escherichia coli infection
Base excision repair
Small cell lung cancer
Aminoacyl-tRNA biosynthesis
Homologous recombination
Hepatitis B
Mismatch repair
Drug metabolism - cytochrome P450
Asthma
Propanoate metabolism
Chemical carcinogenesis
Fatty acid degradation
Valine, leucine and isoleucine degradation
Tyrosine metabolism
Complement and coagulation cascades
Peroxisome
Histidine metabolism
NES
$-log_{10}$(FDR)
C
GI
GII
2646
2589
1821
2573
NKX2-1
RUNX2
D
p=0.0276
p=0.0007
NKX2-1 score
RUNX2 score
E
GroupI
GroupII.1
GroupII.2
F
pcc=0.68,p=5.4e-06
pcc=0.58,p=1.9e-04
Cell cycle score
Embryonic stem cell score
PC1
GI
GII.1
GII.2
G
GII.2 vs GI
GII vs GI
GII.1 vs GI
GII.2 vs GII.1
M value
$-log_{10}$(p-adjust)
log_2(FC)
Overlap with GII.2 vs GI differential peaks
Overlap with GII.2 vs GI DEGs
up
down

Fig. S8

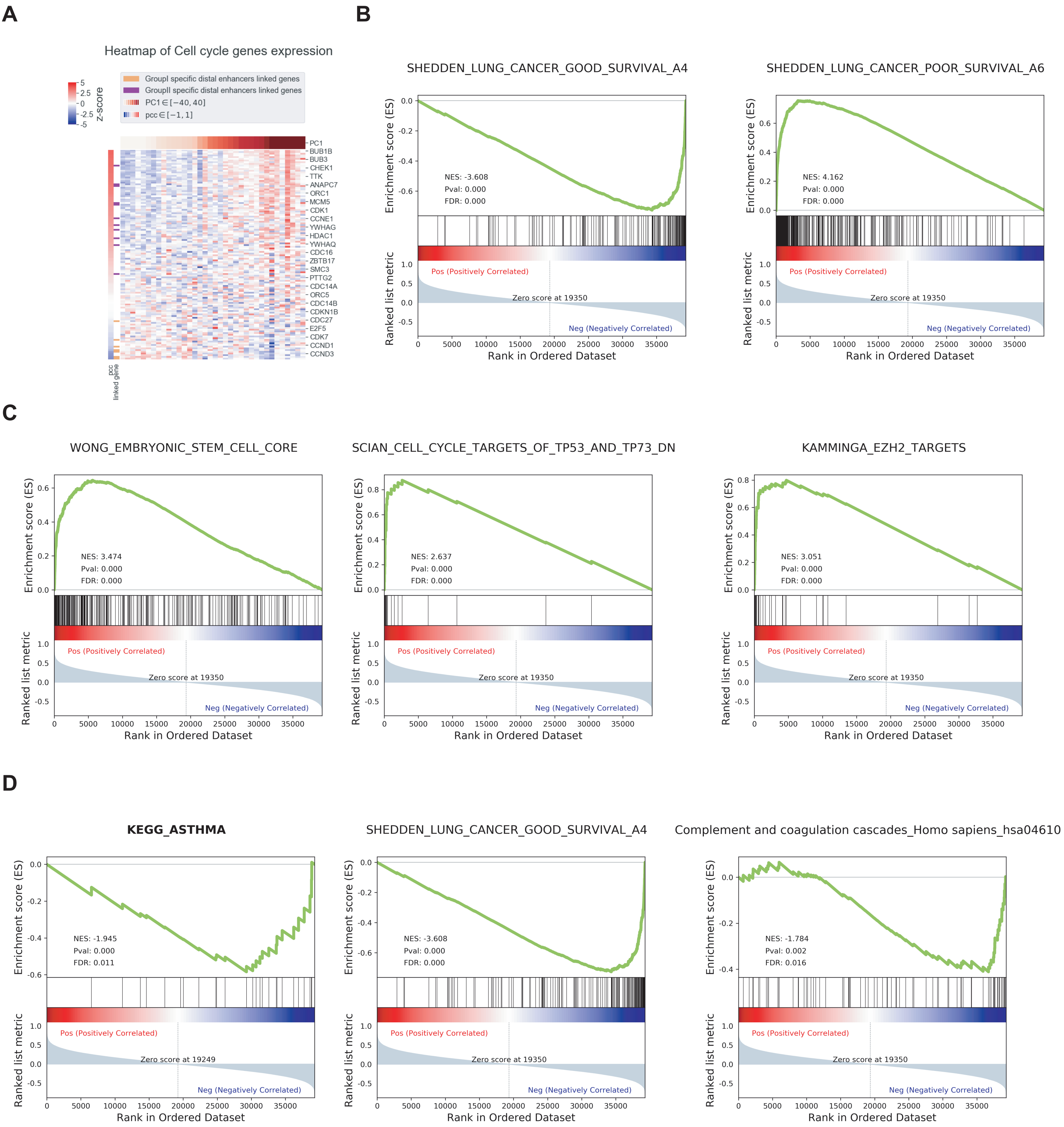
A
B
Heatmap of Cell cycle genes expression
5
2.5
0
-2.5
-5
z-score
GroupI specific distal enhancers linked genes
GroupII specific distal enhancers linked genes
PC1∈[−40, 40]
pcc∈[−1, 1]
PC1
BUB1B
BUB3
CHEK1
TTK
ANAPC7
ORC1
MCM5
CDK1
CCNE1
YWHAG
HDAC1
YWHAQ
CDC16
ZBTB17
SMC3
PTTG2
CDC14A
ORC5
CDC14B
CDKN1B
CDC27
E2F5
CDK7
CCND1
CCND3
pcc
linked gene
SHEDDEN_LUNG_CANCER_GOOD_SURVIVAL_A4
NES: -3.608
Pval: 0.000
FDR: 0.000
SHEDDEN_LUNG_CANCER_POOR_SURVIVAL_A6
NES: 4.162
Pval: 0.000
FDR: 0.000
Enrichment score (ES)
Ranked list metric
Pos (Positively Correlated)
Zero score at 19350
Neg (Negatively Correlated)
Rank in Ordered Dataset
C
WONG_EMBRYONIC_STEM_CELL_CORE
NES: 3.474
Pval: 0.000
FDR: 0.000
SCIAN_CELL_CYCLE_TARGETS_OF_TP53_AND_TP73_DN
NES: 2.637
Pval: 0.000
FDR: 0.000
KAMMINGA_EZH2_TARGETS
NES: 3.051
Pval: 0.000
FDR: 0.000
D
KEGG_ASTHMA
NES: -1.945
Pval: 0.000
FDR: 0.011
Zero score at 19249
SHEDDEN_LUNG_CANCER_GOOD_SURVIVAL_A4
NES: -3.608
Pval: 0.000
FDR: 0.000
Complement and coagulation cascades_Homo sapiens_hsa04610
NES: -1.784
Pval: 0.002
FDR: 0.016

Fig. S9

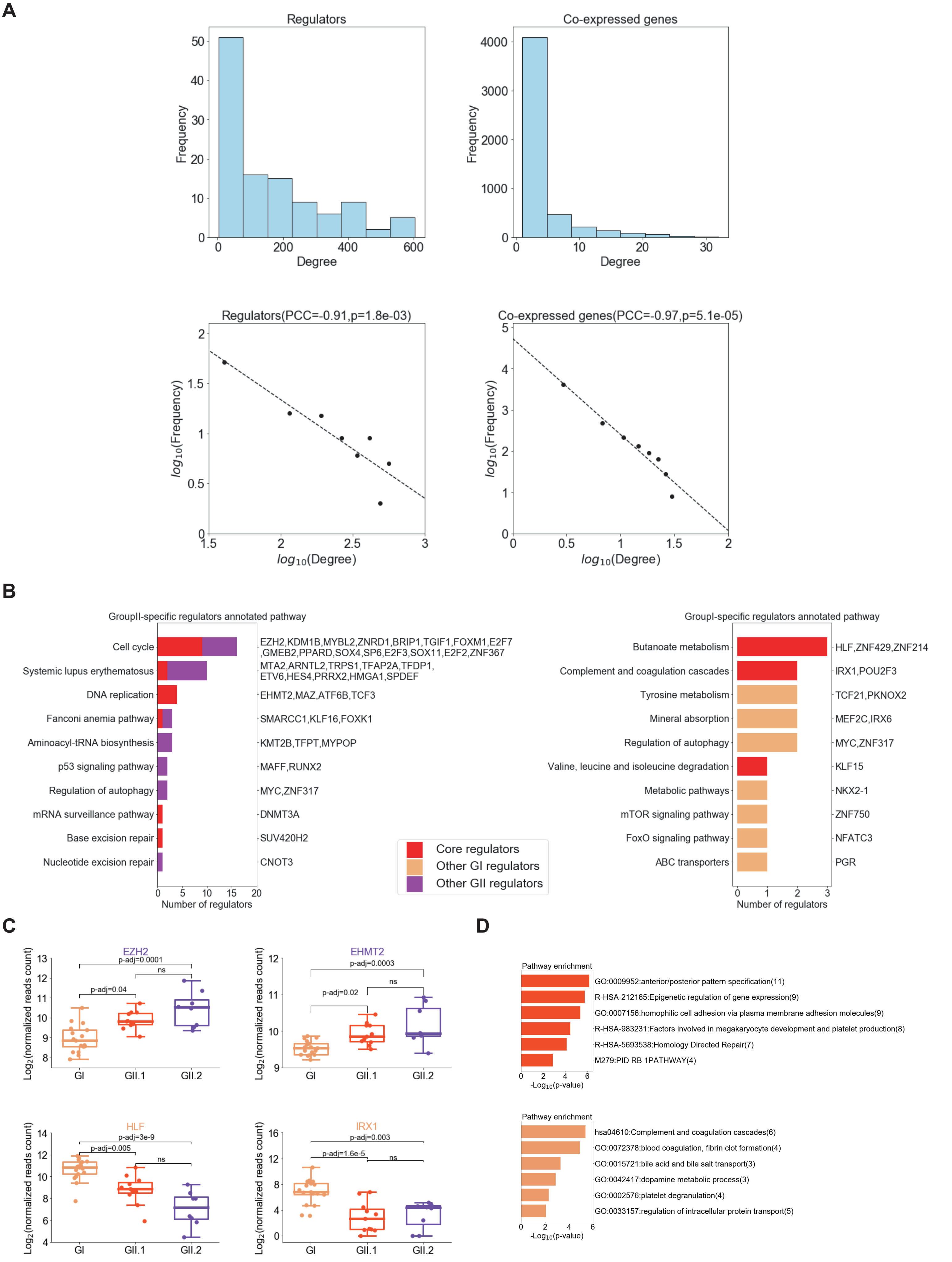
A
Regulators
Frequency
Degree
Co-expressed genes
Frequency
Degree
Regulators(PCC=-0.91,p=1.8e-03)
log_{10}(Frequency)
log_{10}(Degree)
Co-expressed genes(PCC=-0.97,p=5.1e-05)
log_{10}(Frequency)
log_{10}(Degree)
B
GroupII-specific regulators annotated pathway
Cell cycle
Systemic lupus erythematosus
DNA replication
Fanconi anemia pathway
Aminoacyl-tRNA biosynthesis
p53 signaling pathway
Regulation of autophagy
mRNA surveillance pathway
Base excision repair
Nucleotide excision repair
EZH2,KDM1B,MYBL2,ZNRD1,BRIP1,TGIF1,FOXM1,E2F7
,GMEB2,PPARD,SOX4,SP6,E2F3,SOX11,E2F2,ZNF367
MTA2,ARNTL2,TRPS1,TFAP2A,TFDP1,
ETV6,HES4,PRRX2,HMGA1,SPDEF
EHMT2,MAZ,ATF6B,TCF3
SMARCC1,KLF16,FOXK1
KMT2B,TFPT,MYPOP
MAFF,RUNX2
MYC,ZNF317
DNMT3A
SUV420H2
CNOT3
Number of regulators
Core regulators
Other GI regulators
Other GII regulators
GroupI-specific regulators annotated pathway
Butanoate metabolism
Complement and coagulation cascades
Tyrosine metabolism
Mineral absorption
Regulation of autophagy
Valine, leucine and isoleucine degradation
Metabolic pathways
mTOR signaling pathway
FoxO signaling pathway
ABC transporters
HLF,ZNF429,ZNF214
IRX1,POU2F3
TCF21,PKNOX2
MEF2C,IRX6
MYC,ZNF317
KLF15
NKX2-1
ZNF750
NFATC3
PGR
Number of regulators
C
EZH2
p-adj=0.0001
ns
p-adj=0.04
Log_2(normalized reads count)
GI
GII.1
GII.2
EHMT2
p-adj=0.0003
ns
p-adj=0.02
HLF
p-adj=3e-9
p-adj=0.005
ns
IRX1
p-adj=0.003
p-adj=1.6e-5
ns
D
Pathway enrichment
GO:0009952:anterior/posterior pattern specification(11)
R-HSA-212165:Epigenetic regulation of gene expression(9)
GO:0007156:homophilic cell adhesion via plasma membrane adhesion molecules(9)
R-HSA-983231:Factors involved in megakaryocyte development and platelet production(8)
R-HSA-5693538:Homology Directed Repair(7)
M279:PID RB 1PATHWAY(4)
$-Log_{10}$(p-value)
Pathway enrichment
hsa04610:Complement and coagulation cascades(6)
GO:0072378:blood coagulation, fibrin clot formation(4)
GO:0015721:bile acid and bile salt transport(3)
GO:0042417:dopamine metabolic process(3)
GO:0002576:platelet degranulation(4)
GO:0033157:regulation of intracellular protein transport(5)
$-Log_{10}$(p-value)

Fig. S10

A B

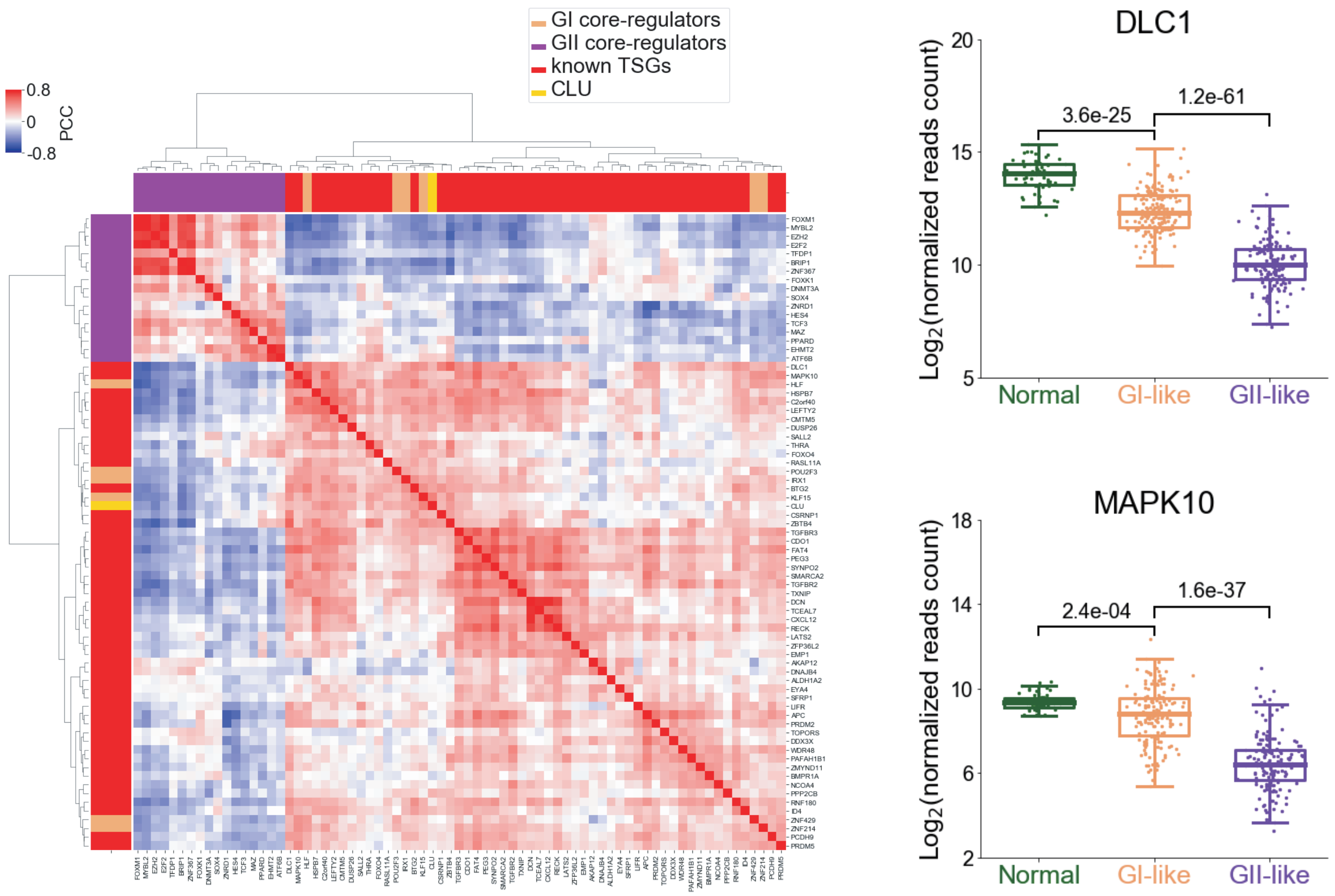
GI core-regulators
GII core-regulators
known TSGs
CLU
0.8
0
-0.8
PCC
DLC1
20
15
10
5
Log2(normalized reads count)
3.6e-25
1.2e-61
Normal
GI-like
GII-like
MAPK10
18
14
10
6
2
2.4e-04
1.6e-37
Normal
GI-like
GII-like

Fig. S11

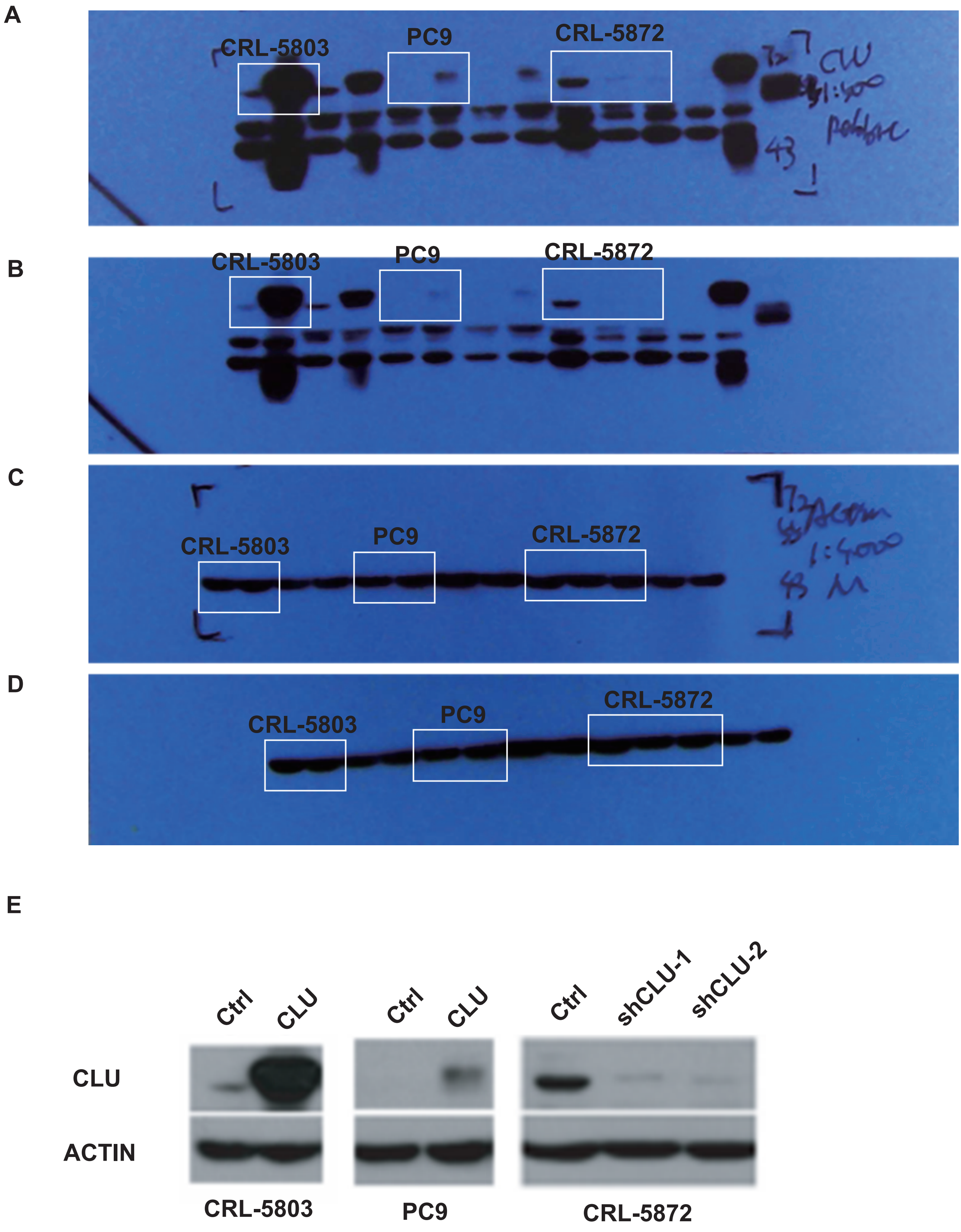
A
CRL-5803
PC9
CRL-5872
B
CRL-5803
PC9
CRL-5872
C
CRL-5803
PC9
CRL-5872
D
CRL-5803
PC9
CRL-5872
E
Ctrl
CLU
Ctrl
CLU
Ctrl
shCLU-1
shCLU-2
CLU
ACTIN
CRL-5803
PC9
CRL-5872

Supplementary Figure Legends

Fig.S1: Cohort analysis of H3K27ac profiles in LUAD tumor and normal tissues.

A) Saturation analysis showing the number of discrete peaks (left) and the number of new peaks (right) with increasing sample size, indicating our epigenetic profiling adequately captured the LUAD patients' epigenome landscape. **B)** Principle component analysis (PCA) based on H3K27ac signal in tumor and normal tissues, two tissues are well separated, three tumor samples locate between normal tissues and other tumor tissues. **C)** The distribution of the genomic location of tumor-specific sites, normal-specific sites and all sites. **D)**Functional enrichment of tumor or normal-specific sites-associated genes. The numbers represent the number of overlap genes within each pathway. **E)** Unsupervised hierarchical clustering of primary tumor tissues and cell lines based on Pearson's correlation coefficient (PCC) calculated via normalized H3K27ac signal in all peaks covered sites. The H3K27 landscapes between cell lines and primary tumors were quite different. **F)** The number of SEs (left) and TEs (right) identified per sample in normal and tumor tissues. **G)** The percentage of SE domains and H3K27ac signals associated with SEs across all samples. The SEs and normalized H3K27ac signals were identified and calculated using the ROSE algorithm.

Fig.S2: Identification of differential SEs.

A) A bar plot showing the definition and the number of four different types of SEs. Rare SEs contained both normal-specific and tumor-specific sites. **B)** A scatter plot demonstrating the fraction of tumor-specific sites within each SE decreased with the increasing of the number of bins within each SE. We used Fisher's exact test to identify differential SEs based on the relative enrichment of differential H3K27ac sites compared to the background. The color bar indicates significance determined by Fisher's exact test. **C)** Box plots indicating the H3K27ac signal change within three different groups of SEs showed similar tendency between tumor and normal tissues. **D)** Differential sites and non-differential sites distributions between SE and TE regions, Fisher-exact test showed differential sites were highly enriched in super-enhancer regions compared with typical-enhancer.

Fig.S3: Linking SEs to genes.

A) A track plot demonstrating the difficulty in assigning genes to SEs, two genes *MAX* and *FUT8* are the super-enhancer proximal gene, H3K27ac signal in promoter of *MAX* were not increased with the H3K27ac signal in the SE, the signal in promoter of *FUT8* was positive correlated with the signal in the SE, thus we provide a more reasonable strategy to assign SE target gene. **B)** A model outlining SE target gene assignment. We assign SE target genes based on Pearson's correlation coefficient between SE-score and gene promoter signal (within 500kb). **C)** The correlation between the SE score and promoter H3K27ac signals of the SEs nearby genes (within 500 kb) across all samples, *FUT8* promoter had significant positive correlation with the SE-score, "*" represented significant signal intensities differences between tumor and normal samples, t-test was used to determine the differences. **D)** A bar plot showing the number of genes mapped per SE, a gene with adjusted p-value less to 0.01 was defined as target gene of the SE. **E)** IHC staining of top-ranked *SIX1*, *SOX4* and *RUNX1* all indicated higher expression of these TFs in tumors. **F)** Track plots of the H3K27ac signal distribution and gene expression in fusion and non-fusion samples across the *SLC34A2* and *ROS1* loci. Number of junction reads from RNA-seq supported *SLC34A2* and *ROS1* gene fusion shown in the left panel.

Fig.S4: Analysis on hyper-variable peaks.

A) The hyper-variable peaks (HVPs) in normal samples identified based on the global trend of means and variances. The dots are colored according to the significance of the variance test performed by MAnorm2, variable peaks with p-value less to 0.01 defined as normal hyper-variable peaks. **B)** A histogram showed the distribution of the expected number of GWAS-SNPs generated from 1,000 random simulations across all peaks, the red line represents the observed number of GWAS-SNPs in hyper-variable peaks identified in tumor samples. **C)** The overlap between tumor hyper-variable peaks(hyper-variable peaks identified in tumor samples), normal hyper-variable peaks(hyper-variable peaks identified in normal samples), peaks upregulated in tumor samples compared to normal samples(previous identified tumor-specific peaks) and peaks upregulated in normal

samples compared to tumor samples(previous identified normal-specific peaks). **D)** Permutation was used to identify significant PCs among hyper-variable peaks in tumor. **E)** The frequent driver gene mutation states of 42 LUAD samples by group. The mutation frequency of *P53* was higher in Group II (58.8%) compared with Group I (36.8%), but the difference was not significant. Three *EML4-ALK* fusion samples were all classified into Group II, one *SLC34A2-ROS1* fusion sample was classified into Group I.

Fig.S5: Comparison of clustering results based on ChIP-seq and RNA-seq

A) Consensus matrix resulting from consensus clustering analysis based on principle component 1 of tumor hyper-variable peaks. k=2 classified the tumor samples into Group I (GI) and Group II (GII). k=3 classified the tumor samples into Group I (GI), Group II.1 (GII.1) and Group II.2 (GII.2). The strength of the blue color is proportional to the frequency at which samples have been clustered together. **B)** Using Limma-Trend to identify hyper-variable genes (p-value less to 0.05) in tumor samples RNA-seq data. **C)** Consensus matrix resulting from consensus clustering analysis based on principle component 1 of hyper-variable genes. **D)** Comparison of the clustering results based on ChIP-seq (k=2) and RNA-seq (k=2) (p-value of Fisher exact test indicated the consistency of two clustering results). **E)** Survival analysis of the classification result of ChIP-seq (k=2) and RNA-seq (k=2) : Relapse-free survival (top) and Overall survival (bottom), p-value of log rank test showed in the plot, we have control the sample size in order to make p-value comparable.

Fig.S6: Assigning enhancers to genes.

A) A model showing how the distal enhancers (or SEs) were linked to genes via the correlation of H3K27ac signals in enhancer and gene expression within 500kb. **B)** The distribution of non-specific PCC that was used to calculate the p-value of PCC, non-specific PCCs were calculated via the PCC of randomly select 100,000 genes (not within 500kb or not in the same chromosome) and enhancer pairs. The mean and standard deviation for these non-specific correlations were used to calculate the significance of PCC. **C)** The distribution of the distances from distal enhancers to the TSSs of its linked genes, the number of significant links decrease rapidly with the increased distance. 500kb was

effective to identify significant linked genes. **D)** The distribution of the number of genes mapped per enhancer, most of enhancers were mapped to one gene. **E)** The distribution of enhancers mapped per gene, most of genes were linked to more than one enhancer. **F)** The distribution of the number of skipped genes, about half of links skipped over one or more genes.

Fig.S7: Epigenetic signatures in GI/GII and the relation to transcriptomic features.

A) Box plots in the top panel indicating the H3K27ac signal changes showed same tendency within three different groups of SEs. Box plots in the bottom panel indicating the gene expression level changes within three different groups of SE-associated genes was highly associated with epigenetic change. **B)** GSEA based on the correlation of PC1 and gene expression level revealed different signatures in GI (right) and GII (left). **C)** IHC staining showed the down regulation of *NKX2-1* and up-regulation of *RUNX2* in GII samples (1821 and 2573) compared with GI samples (2646 and 2589) **D)** Scatter plot showed the IHC score of *NKX2-1* and *RUNX2* in GI and GII samples. *NKX2-1* was significantly down regulated and *RUNX2* was up regulated in GII sample. **E)** LUAD samples were further clustered into three subgroups based on previous hierarchical clustering result. GII was further divided into GII.1 and GII.2. **F)** Scatter plots showing the correlation between PC1 and the ssGSEA-score of two pathway related to stem cell, stemness increasing during the progression in PC1. A gradually up-regulation of activities in stem cell related pathways were observed across GI, GII.1 and GII.2. **G)** Epigenomic(top) and transcriptomic(bottom) differential analysis results between GI, GII.1 and GII.2. Volcano plot showing the differences among the subgroups, GI and GII.1 is quite different on epigenome but similar in transcriptome compared to the differential analysis results between GII.2 and GI. GII.1 is similar to GII.2 in both epigenome and transcriptome.

Fig.S8: Important biological pathways uncovered by epigenetic signatures.

A) Heatmap of cell cycle pathway genes expression across tumor samples, genes ranked by pearson correlation coefficient between genes expression and PC1 in hyper-variable peaks. Most of cell cycle genes were positive correlated with PC1 and 18 of 124 cell cycle

genes were linked to group-specific distal enhancers. **B)-D)** Important biological pathways identified by GSEA using the ranked PCC between gene-expression and PC1. **B)** Our epigenetic classification model can be verified by another cohort analysis based on LUAD transcriptome. **C)** Important pathways enriched in GII, embryonic stem cell core indicated GII was stem cell like tumor, cell cycle targets of *TP53* and *TP73* down indicated cell cycle genes up-regulation might related to *TP53* and *TP73*, *EZH2* targets indicated alterations in GII might relate to epigenetic regulators. **D)** Important pathway enriched in GI, suggested GI maintained some normal like function and immune-cell related signaling pathways.

Fig.S9: Core regulators identification and the functional signature.

A) The distribution of degree of regulators (left 2 panels) and co-expressed genes (right 2 panels) were in line with a power law form. **B)** The functional signatures of GI-specific regulators (right) and GII-specific regulators (right), core regulators(red) were annotated to top ranked biological pathways. Regulators were annotated by the most significant pathway enriched based on its co-expressed genes. Pathways were ranked by the number of regulators annotated to this pathway. **C)** Expression changes of four group-specific core-regulators across GI, GII.1 and GII.2. Two GII-specific core regulators and two GI-specific core regulators identified as GII.1 up-regulated genes(n=62) and down-regulated genes(n=61) compared to GI were showed in the up panel and bottom panel respectively, adjusted p-value calculated via DESeq2. These Group-specific core-regulators gradually upregulated or downregulated across GI, GII.1 and GII.2. **D)** Function enrichment of GII.1 up-regulated genes and down-regulated genes, epigenetic regulation of gene expression was firstly altered between GI and GII.1.

Fig.S10: The relationship between core regulators and TSGs.

A) Pearson correlation matrix for core-regulators and TSGs of TCGA-LUAD cohort. *CLU* was positive correlated with GI-specific core-regulators but negative correlated with GII-specific core-regulators. **B)** Gene expression level of *MAPK10* and *DLC1* in normal, GI-like and GII-like samples in TCGA-LUAD cohort, p-value of t-test was showed in the plot.

Fig.S11:The full uncropped Western blot picture and related separate picture in Figure 5.

A-B) Uncropped Western blot detection of *CLU* in CRL-5803, PC9 and CRL-5872 cells with ectopic expression, knockdown or without treatment under long(A) and short(B) time exposure. **C-D)** Uncropped Western blot detection of *Actin* in CRL-5803, PC9 and CRL-5872 cells with ectopic expression, knockdown or without treatment under long(C) and short(D) time exposure. **E)** Related Western blot detection results of *CLU* and *Actin* in Figure 5.
