# Supplementary material for: A systematic dissection of the epigenomic heterogeneity of lung adenocarcinoma reveals two different subclasses with distinct prognosis and core regulatory networks
Source: Genome Biol. 2021 May 17;22:156. doi: 10.1186/s13059-021-02376-1 (PMC8127276; doi:10.1186/s13059-021-02376-1)
Supplement: Supplementary file 12 — Additional file 12. Review history. [file 13059_2021_2376_MOESM12_ESM.docx]

Review History

**First round of review**

**Reviewer 1**

**Are you able to assess all statistics in the manuscript, including the appropriateness of statistical tests used?** Yes, and I have assessed the statistics in my report.

**Comments to author:**

Overall Summary:

Yuan et al. investigated the H3K27ac histone modification profiles of the tumor and adjacent normal lung tissues from 42 LUAD patients and explored the role of epigenetic alterations in LUAD progression. They identified inter-tumor variations of H3K27ac levels at proximal gene promoters and distal enhancers and clustered the LUAD patients into two different groups based on their H3K27ac signals. Then the authors identified the super-enhancers or core regulators based on co-expression analysis for group I and group II LUAD patients. They found GII constituted a high-risk pathological subgroup and was composed of patients with micro-papillary and solid predominant LUAD. By contrast, GI contained more low-risk pathological subgroup patients. From the distal enhancer and core-regulator analysis, the authors identified CLU as a group I specific core regulator and validated it as a tumor suppressor gene in vivo in KP mouse. However, the rationale for why cluster the patients into two groups needs to be clarified. Most of the figures are not well presented and with low resolution. Most of the pathways enriched by either the SE associated genes from the H3K27ac profiles or the DEGs from the RNA-seq profiles of LUAD are not oncogenic pathways, thus these results seem unconvincing. A more deep explanation about how the group-specific enhancers or super-enhancers contribute to lung tumorigenesis or progression needs to be clarified or validated by experimental approaches, at least pathological staining. It may provide valuable insight if the following concerns are addressed.

Major Comments:

1. The resolution of many figures is too low and they must be adjusted. For example, Supplementary Figure 7A, Figure 2A, and Figure 2B. The font size of the text in those figures is too small.

2. Not sure why in Fig 1A, 3 LUAD patients are clustered together with normal samples.

3. Fig 1F listed out a lot of enriched pathways or GO terms, not sure any of those GO terms or pathways are correlated with LUAD tumorigenesis or progression.

4. It seems that the authors identified 4,615 tumor hyper-variable H3K27ac peaks (HVPs). What's the corresponding cutoff? How many normal hyper-variable peaks are there? Could the authors provide those HVP regions in supplementary materials?

5. "We found that most HVPs were not tumor-specific or normal hyper-variable peaks (Figure 2.B).", I can't figure out why those HVPs are tumor-specific or not? What kind of method did the authors use to determine an HVP is tumor-specific or not?

6. Some of the figures are not self-explanatory. For example, for Figures 1G and 1H, even with illustration, I still cannot figure out what is the peaks of CAV2CAV1 and MET gene indicates. It seems like the super-enhancer of EML4 exists in both LUAD and normal samples, and the SE of CAV2CAV1 only exists in the normal sample.

7. Why cluster the 42 LUAD patients into two groups, not three or four groups? From Figure 2D, clearly, group II is consists of two subgroups, and these two sub-groups of GII show different pathological subtypes, tumor size, and lymph node metastasis status.

8. "3 of 42 patients were detected ALK-EML4-positive in our study, and we found that the hijacked SEs were key regulators that maintained ALK-fusion gene expression in all three patients (Figure 1-H). The same result was also found in one ROS1-SLC34A2-positive patient (Supplementary Figure 2-E)." Please define hijacked SE and make it understandable to readers. Besides, it is difficult for the reviewer to connect the results of Figures 1H and 2E to the authors' conclusion. A more detailed explanation needs to be added.

9. "After a quantitative comparison of the GI and GII H3K27ac profiles…", not sure what kind of comparison approach is employed? Would the authors write down their detailed methods?

10. As shown in Figure 3B, the group II DEGs only enriched with two relevant oncogenic pathways, i.e., the cell cycle and DNA replication, and also enriched with normal development pathways, making the results unconvincing.

11. "The genes upregulated in GI tend to be associated with maintaining normal cell function, and are enriched in metabolic processes". These results are very confusing, seems like there are no dysfunctional oncogenic pathways enriched from the DEGs of group I LUAD.

12. Figures 4A and 4B need to be adjusted to make them more clear. Besides, what's the meaning of 'ER' in these figures? It is very confusing.

13. In my opinion, the authors think distal enhancers and SEs are the same according to Figures 5A and 5B?

14. What is the definition of core-regulators? How to get those core-regulators?

15. In line 4 of the Method section, did the authors consider the "other effects" in the DESeq2 model? Please clarify the method of how to identify DEGs from the RNA-seq profiles of LUAD patients.

16. I suggest adding tumor purity estimation of the LUAD samples, as it seems like that the enriched pathways got from the H3K27ac data are very different from the RNA-seq data.

17. "This finding emphasizes that SE abnormalities might be crucial factors in tumorigenesis." Experiments such as pathological staining need to be done to show if those super-enhancers or SE associated transcription factors are correlated with LUAD tumorigenesis or progression.

18. 'Based on the literature and RNA-seq data, we noticed CLU and PLA2G1B, two GI-specific genes which were both included in our previous TSGs screening study.' I am still not sure why the authors select CLU as the top-ranked TSG and validate it in-vivo, the rationale needs to be clarified. Would the authors rank all the TSG candidates?

19. In Figures 5D and 5E, the authors showed the mouse model results. Knocking out CLU increased the lung tumor burden in tumor recipient mice. However, did the authors inject the group I patient-derived tumor cells into the recipient mice? Why the recipient mouse develop the group I type LUAD? H&E, Ki-67, and CLU staining should be added to convince the role of CLU as a tumor suppressor in Group I LUAD.

20. In line 53 of the Discussion section. The authors concluded that GII is transformed from GI at an early stage. How did they get the temporal relationship between GII and GI? Did they conduct trajectory analysis to demonstrate that? More late-stage samples in GII than GI doesn't mean that GII and GI have the same tumor progenitors.

Minor Comments:

1. Abbreviations need to be spelled out or defined when they first appear. For example, PFS and OS.

2. There are many syntax errors. For example, in line 52, 'By contrast, the tumor-specific sites are highly enriched in transcriptional regulation, indicating that aberrant epigenetic modification serves dysregulate transcription in LUAD (Figure 1-D).'; 'These data suggest that SE abnormality directly get involved in LUAD oncogenesis by regulating driver genes. Taken together, the results underscored the role of SE in LUAD as key regulators.'; 'When perform survival analysis in each stage, we found that GI-GII score can identify high-risk patients in stage I cohort (Figure 3-H).'; 'Differential epigenetic element acted as an upstream influenced the expression of these core regulators.'

**Reviewer 2**

**Are you able to assess all statistics in the manuscript, including the appropriateness of statistical tests used?** Yes, and I have assessed the statistics in my report.

**Comments to author:**

In this study, Yuan et al. applied the histone modification H3K27ac to facilitate the tumor-subtyping of lung adenocarcinoma. First, the authors showed the global H3K27ac profile in tumor and normal samples, and the differential peaks are related to transcription dysregulation in LUAD. The super enhancer-associated genes are also related to oncogenesis. In addition, fusion gene expression is correlated with abnormality of super enhancer activity. Then they quantified the variation of H3K27ac signals in tumor samples, and termed it as the 'intertumor epigenomic heterogeneity'. Two LUAD subgroups(GI and GII) were classified using the most variable H3K27ac peaks, and they have distinct pathological risk level and survival rate. Based on the subgroup specific genes, they reanalyzed LUAD data in TCGA cohort and defined GI-like and GII-like patients. And the survival rate and mutation frequency of these two groups of patients are different. Furthermore, the regulating networks of GI and GII-specific genes are distinct. Finally, the authors came up with a candidate gene, CLU, which is lower expressed in GII but not GI. And the knock-out experiments suggest it may serve as a TSG. Overall, this study expanded our understanding of tumor heterogeneity from the perspective of histone modification. But some description and definitions are not clearly presented. Before considered as suitable for publication, some concerns need to be clarified first.

* Recently, two Cell papers revealed the molecular signatures of lung cancer mainly based on proteomics. What's the benefit of H3K27ac-based subtyping compared to RNA or proteomic subtyping? Whether it can better explain survival or pathological risk?

* The legend of Fig1A is not correct. The clustering is based on the Pearson's correlation coefficient (PCC), but not H3K27ac signals.

* What does the H3K27ac profile at ALK and EML4 look like in ALK-EML4 fusion samples? Any difference from non-fusion samples?

* For the PCA analysis, what is the percentage of each PC?

* The authors showed that H3K27ac-based subtypes present different mutation frequency. Whether the regional mutation rate is associated with H3K27ac variation level?

* The authors should explain how the GII group was further divided to GII.1 and GII.2. I cannot find any description or threshold.

* The definition of GI-like and GII-like patients are based on similarity of transcriptomes. But gene expression is not fully determined by H3K27ac, so such classification may or may not related to H3K27ac profile.

* The authors claimed 'higher epigenomic heterogeneity in GII than GI'. But they haven't specify which part of results can support it.

* The figures and legends need re-organization. Some of them are not explained in main text, like Fig1G. And I don't know why the 'chrY' are highlighted in Fig2A. And the order of some figures are not consistent with text.

**Authors Response**

**Point-by-point responses to the reviewers’ comments:**

**Reviewer #1: Overall Summary:**
*Yuan et al. investigated the H3K27ac histone modification profiles of the tumor and adjacent normal lung tissues from 42 LUAD patients and explored the role of epigenetic alterations in LUAD progression. They identified inter-tumor variations of H3K27ac levels at proximal gene promoters and distal enhancers and clustered the LUAD patients into two different groups based on their H3K27ac signals. Then the authors identified the super-enhancers or core regulators based on co-expression analysis for group I and group II LUAD patients. They found GII constituted a high-risk pathological subgroup and was composed of patients with micro-papillary and solid predominant LUAD. By contrast, GI contained more low-risk pathological subgroup patients. From the distal enhancer and core-regulator analysis, the authors identified CLU as a group I specific core regulator and validated it as a tumor suppressor gene in vivo in KP mouse. However, the rationale for why cluster the patients into two groups needs to be clarified. Most of the figures are not well presented and with low resolution. Most of the pathways enriched by either the SE associated genes from the H3K27ac profiles or the DEGs from the RNA-seq profiles of LUAD are not oncogenic pathways, thus these results seem unconvincing. A more deep explanation about how the group-specific enhancers or super-enhancers contribute to lung tumorigenesis or progression needs to be clarified or validated by experimental approaches, at least pathological staining. It may provide valuable insight if the following concerns are addressed.*

**Response in summary：**

We thank this reviewer for her/his critical and insightful comments. In the revision, we have made changes to solve the corresponding concerns. First, we used the ConsensusClusterPlus algorithm to evaluate the stability of clustering results under different cluster numbers (Supplementary Figure 5A) and added a detailed description of this analysis in the section of “Methods” in the revised manuscript. Second, we increased the font size of text and the resolution of figures in the revised manuscript. Third, we used GSVA to evaluate the activity of enriched gene sets and proved a general association between the enriched pathways/terms and LUAD tumorigenesis or progression. Fourth, we added experiments of IHC staining of serval tumor-specific and subgroup-specific SE associated transcription factors to investigate the role of these transcription factors in LUAD tumorigenesis and progression as this reviewer suggested. We hope this reviewer will find the manuscript much improved.

**Major Comments:**

**Q1.** *The resolution of many figures is too low and they must be adjusted. For example, Supplementary Figure 7A, Figure 2A, and Figure 2B. The font size of the text in those figures is too small.*

**Response 1:**

Sincerely thanks for pointing out this issue. We have increased the font size of text and the resolution of figures in the revised manuscript.

**Q2.** *Not sure why in Fig 1A, 3 LUAD patients are clustered together with normal samples.*

**Response 2:**

Thanks for pointing out this issue. We believe the primary reason is that the 3 LUAD patients were in very early tumor stages and their tumor tissues were less malignant compared to the other LUAD patients. For example, one of the 3 LUAD patients (2469) had the minimally invasive adenocarcinoma among all the 42 patients, and another one (2589) had a GGO-type LUAD. Consistently, in our classification model the 3 tumor samples had been classified to GI, which was associated with better clinical outcome and earlier tumor stage than GII. We further performed principle component analysis (PCA) on H3K27ac profiles of all the tumor and normal tissues (Supplementary Figure 1-C). It can be seen that the two types of tissues are well separated from each other and the 3 tumor profiles are more of an intermediate status.

**Q3.** *Fig 1F listed out a lot of enriched pathways or GO terms, not sure any of those GO terms or pathways are correlated with LUAD tumorigenesis or progression.*

**Response 3:**

Thanks for pointing out this issue. This is a very good point about the interpretation of biological pathways and GO terms. Firstly, we want to stress that two genes involved in the same pathway (or GO term) could play different and even opposing roles for LUAD tumorigenesis or progression, which explains why some pathways/terms have been enriched from target genes of tumor-specific SEs as well as from target genes of normal SEs (e.g., regulation of cell adhesion and regulation of protein kinase activity; Figure 1-G in the revised manuscript). For example, DLC1, which is a target gene of normal SEs, is involved in the GO term of regulation of cell adhesion (Figure 1-E; Supplementary Table 9). This gene is a well-known LUAD suppressor gene, which exerts its strong suppressive function by positively regulating adherens-junction stability [1]. By contrast, SOX9, which is a target gene of tumor SEs and is involved in the same GO term (Figure 1-E; Supplementary Table 10), plays an opposing role in regulating cell adhesion and can thus promote LUAD progression [2,3].

Therefore, for a more accurate evaluation of the enriched pathways and GO terms, we have taken the overlap between the genes associated with each pathway/term and the genes from which the pathway/term was enriched (i.e., target genes of tumor-specific SEs or target genes of normal-specific SEs). We then used GSVA [4] to assess the activity of each overlapping gene set in each patient of the TCGA-LUAD cohort (based on the corresponding RNA-seq data set). It can be seen that all the gene sets are associated with significantly different activity between normal and tumor tissues, and the relative activity of each gene set was consistent with the relative activity of SEs (Figure R1-A). Besides, the activity of most of the tumor gene sets was well correlated with lymph node invasion status in LUAD patients (Figure R1-B). Altogether, these results suggested a general association between the enriched pathways/terms and LUAD tumorigenesis or progression.

**References:**

1.	Tripathi V, Popescu NC, Zimonjic DB: **DLC1 Interaction with alpha-Catenin Stabilizes Adherens Junctions and Enhances DLC1 Antioncogenic Activity.** *Molecular and Cellular Biology* 2012, **32:**2145-2159.

2.	Francis JC, Capper A, Ning J, Knight E, de Bono J, Swain A: **SOX9 is a driver of aggressive prostate cancer by promoting invasion, cell fate and cytoskeleton alterations and epithelial to mesenchymal transition.** *Oncotarget* 2018, **9:**7604-7615.

3.	Laughney AM, Hu J, Campbell NR, Bakhoum SF, Setty M, Lavallee VP, Xie YB, Masilionis I, Carr AJ, Kottapalli S, et al: **Regenerative lineages and immune-mediated pruning in lung cancer metastasis.** *Nature Medicine* 2020, **26:**259-+.

4.	Hanzelmann S, Castelo R, Guinney J: **GSVA: gene set variation analysis for microarray and RNA-Seq data.** *Bmc Bioinformatics* 2013, **14**.

**Q4.** *It seems that the authors identified 4,615 tumor hyper-variable H3K27ac peaks (HVPs). What's the corresponding cutoff? How many normal hyper-variable peaks are there? Could the authors provide those HVP regions in supplementary materials?*

**Response 4:**

Sincerely thanks for pointing out this issue. For the identification of tumor and normal HVPs, we used the varTestBioCond function in the MAnorm2 package (https://github.com/tushiqi/MAnorm2), which fits a smoothed curve on mean-variance scatter plot (e.g., Figure 2-A) and assesses the statistical significance of each peak based on how deviated it is from the curve (in the scatter plot). We used a p-value cutoff of 0.01, and identified 4,615 and 2,373 HVPs for tumor and normal samples, respectively (Figure 2-A and Supplementary Figure 4-A). All these HVPs are provided in Supplementary Table 7 and Supplementary Table 8.

**Q5.** *"We found that most HVPs were not tumor-specific or normal hyper-variable peaks (Figure 2.B).", I can't figure out why those HVPs are tumor-specific or not? What kind of method did the authors use to determine an HVP is tumor-specific or not?*

**Response 5:**

We apologize for the misleading text. We agree that the sentence can easily lead to confusion. “Most HVPs” in the sentence refers to HVPs identified in tumor samples. “tumor-specific peaks” refers to the identified differential peaks that are up-regulated in tumor samples compared to normal samples. We did not determine whether each HVP was tumor-specific or not. Instead, we identified HVPs separately in tumor samples and normal samples, and found the overlap between tumor HVPs and normal HVPs was small. We have corrected the misleading text in the revised manuscript as follows: “We further noticed that the vast majority of the tumor HVPs were not identified as HVPs across normal samples (Figure 2B; Supplementary Figure 4C). It indicates that most of the intertumoral epigenetic heterogeneity observed at the tumor HVPs can hardly be explained by the epigenetic variations already exist in the patients’ normal lung tissue and it predominantly emerged during tumorigenesis. Meanwhile, there was little overlap between the tumor HVPs and the peaks upregulated in tumor samples compared to normal samples (previous identified tumor-specific peaks) showing consistent H3K27ac changes between tumor and normal tissues (Figure 2B), suggesting that a very large fraction of them may be peaks specific to a subset of the tumor samples. Thus, further investigation on the observed inter-tumor epigenetic heterogeneity is needed and may provide insights into LUAD progression” (Page 8).

**Q6.** *Some of the figures are not self-explanatory. For example, for Figures 1G and 1H, even with illustration, I still cannot figure out what is the peaks of CAV2CAV1 and MET gene indicates. It seems like the super-enhancer of EML4 exists in both LUAD and normal samples, and the SE of CAV2CAV1 only exists in the normal sample.*

**Response 6:**

We apologize for the confusion. In Figure 1G in the original manuscript, we intended to use examples to illustrate the role of SEs in LUAD progression. Specifically, for examples of up-regulated SEs in tumor samples, we selected those SEs associated with SOX9, which is a well-known regulator that can promote LUAD progression [1]. For down-regulated SEs in tumor samples, we used the SEs associated with CAV1 and CAV2, which are marker genes of normal lung alveolar cells [2]. For reference, we also showed an SE that was not associated with significantly differential H3K27ac levels between tumor and normal samples (i.e., the SE associated with MET). We agree that the original manuscript did not provide a clear explanation of this figure, and we have added a detailed description in the revised manuscript (Page 7 and Figure 1-H).

In Figure 1H in the original manuscript, we used an example to illustrate “super-enhancer hijacking”, which is a specific type of genome translocation in which a super enhancer is translocated to another genomic region. For a better graphical presentation, we refined Figure 1H (Figure 1-I in the revised manuscript) as follows: RNA-seq junction reads that suggest gene fusion of *EML4* and *ALK* are shown in the top left panel; an illustration model of super-enhancer hijacking through gene fusion is shown in the top right panel; tracks for H3K27ac and gene expression levels at *EML4* and *ALK* loci in example fusion and non-fusion tumor samples are shown in the bottom panel. As we can see, the expression level of ALK is clearly much higher in the fusion sample than that in the non-fusion sample.

**References:**

1.	Laughney AM, Hu J, Campbell NR, Bakhoum SF, Setty M, Lavallee VP, Xie YB, Masilionis I, Carr AJ, Kottapalli S, et al: **Regenerative lineages and immune-mediated pruning in lung cancer metastasis.** *Nature Medicine* 2020, **26:**259-+.

2.	LaFave LM, Kartha VK, Ma S, Meli K, Del Priore I, Lareau C, Naranjo S, Westcott PMK, Duarte FM, Sankar V, et al: **Epigenomic State Transitions Characterize Tumor Progression in Mouse Lung Adenocarcinoma.** *Cancer Cell* 2020, **38:**212-+.

**Q7.** *Why cluster the 42 LUAD patients into two groups, not three or four groups? From Figure 2D, clearly, group II is consists of two subgroups, and these two sub-groups of GII show different pathological subtypes, tumor size, and lymph node metastasis status.*

**Response 7:**

Sincerely thanks for raising this important point. We have actually used the ConsensusClusterPlus algorithm [1] to evaluate the stability of clustering results under different cluster numbers (referred to as k). Specifically, clustering results from k=2 are clearly more stable than k=3 (Supplementary Figure 5-A; we have also added a detailed description of this analysis in Methods in the revised manuscript; Page 16). Considering also the limited cohort size, we have eventually chosen to use k=2 for downstream analysis.

In fact, we have also tried k=3, which has further divided GII into GII.1 and GII.2 (Supplementary Figure 7-E). Indeed, the activity of two stem cell related pathways is significantly different between GII.1 and GII.2 (Supplementary Figure 7-F). However, the overall epigenomic differences between GII.1 and GII.2 are subtle (Supplementary Figure 7-G, last column).

**References:**

1.	Wilkerson MD, Hayes DN: **ConsensusClusterPlus: a class discovery tool with confidence assessments and item tracking.** *Bioinformatics* 2010, **26:**1572-1573.

**Q8.** *"3 of 42 patients were detected ALK-EML4-positive in our study, and we found that the hijacked SEs were key regulators that maintained ALK-fusion gene expression in all three patients (Figure 1-H). The same result was also found in one ROS1-SLC34A2-positive patient (Supplementary Figure 2-E)." Please define hijacked SE and make it understandable to readers. Besides, it is difficult for the reviewer to connect the results of Figures 1H and 2E to the authors' conclusion. A more detailed explanation needs to be added.*

**Response 8:**

We apologize for the unclear descriptions. Super enhancer hijacking is a specific type of genome translocation in which a super enhancer is translocated to another genomic region and establishes a regulatory relationship with new target genes. ALK-fusion and ROS1-fusion are both important driver mutations in LUAD tumorigenesis, and ALK-fusion positive patients could largely benefit from TKIs target-therapy. Previous studies have found various forms of ALK-fusion and the corresponding partner genes, but we first noticed that a super enhancer at upstream of EML4 could be hijacked (through ALK-EML4 fusion) and thus increase the expression level of ALK. The hijacked SE therefore might be a potential therapeutic target for LUAD patients who are TKIs-resistant.

In the revised manuscript, we have given a clear definition of super enhancer hijacking (Page 7). We have also refined the figures for a better graphical presentation (Figure 1-I and Supplementary Figure 3-E in the revised manuscript). As we can see, super-enhancers of highly expressed genes (e.g., *EML4*-SE and *SLC34A2*-SE) can serve as regulatory elements of oncogenes (e.g., *ALK* and *ROS1*) through super enhancer hijacking. We did realize that the connection was weak between these results and the conclusion that SEs are key regulators in LUAD progression, and we have removed the conclusion in the revised manuscript (Page 7).

**Q9.** *"After a quantitative comparison of the GI and GII H3K27ac profiles…", not sure what kind of comparison approach is employed? Would the authors write down their detailed methods?*

**Response 9:**

Thanks for this suggestion. We have actually performed the differential analysis by applying MAnorm2, which is a computational tool specifically developed for quantitatively comparing groups of ChIP-seq samples [1]. Those peaks with an adjusted p-value less than 0.01 were defined as group-specific peaks. In the revised manuscript, we refined the text to “After a quantitative comparison of the H3K27ac profiles between GI and GII tumors using MAnorm2, we defined 17,713 GII-specific and 13,408 GI-specific H3K27ac peaks” (Page 9).

**References:**

1.	Tu S, Li M, Tan F, Chen H, Xu J, Waxman DJ, Zhang Y, Shao Z: **MAnorm2 for quantitatively comparing groups of ChIP-seq samples.** *bioRxiv* 2020**:**2020.2001.2007.896894.

**Q10.** *As shown in Figure 3B, the group II DEGs only enriched with two relevant oncogenic pathways, i.e., the cell cycle and DNA replication, and also enriched with normal development pathways, making the results unconvincing.*

**Response 10:**

Thanks for pointing out this issue. To evaluate the reliability of the GII enriched pathways, we used GSVA [1] to assess the activity of each of them in each patient of the TCGA-LUAD cohort. For each pathway, we then performed a COX proportional hazard ratio regression to assess the correlation between the pathway activity and the survival time of patients. We found that the vast majority of the GII enriched pathways were significantly negatively associated with survival time (p<0.05 and HR>0; Figure R2). The only exception was the pathway of chordate embryonic development (p>0.05 and HR>0). Still, we found several genes in this pathway were known activators of LUAD progression, such as RUNX2 and FOSL1 [2]. Altogether, these results indicated an association between the GII enriched pathways and LUAD progression.

**References:**

1.	Hanzelmann S, Castelo R, Guinney J: **GSVA: gene set variation analysis for microarray and RNA-Seq data.** *Bmc Bioinformatics* 2013, **14**.

2.	LaFave LM, Kartha VK, Ma S, Meli K, Del Priore I, Lareau C, Naranjo S, Westcott PMK, Duarte FM, Sankar V, et al: **Epigenomic State Transitions Characterize Tumor Progression in Mouse Lung Adenocarcinoma.** *Cancer Cell* 2020, **38:**212-+.

**Q11.** *"The genes upregulated in GI tend to be associated with maintaining normal cell function, and are enriched in metabolic processes". These results are very confusing, seems like there are no dysfunctional oncogenic pathways enriched from the DEGs of group I LUAD.*

**Response 11:**

We apologize for the misleading sentence. The point is that the GI enriched pathways were derived from the DEGs between GI and GII rather than between GI and normal tissues. The DEGs up-regulated in GI (or equivalently, down-regulated in GII) tended to be associated with normal lung cell functions (e.g., surfactant metabolism and branched-chain amino acid catabolism; Figure 3-B), which was consistent with the observation that GII was more malignant than GI as well as the assumption that GI might correspond to early-stage tumors and precede GII. Besides, several independent studies have shown that Kras-driven murine LUAD preferentially arises from AT2 cells (alveolar type II cells), which has also been supported by the immunohistochemical staining of alveolar markers in early-stage tumors [1,2]. Consistently, canonical AT2 markers such as surfactant proteins (SFTPC, SFTPA1) were associated with significantly higher expression levels in GI than in GII [2]. Moreover, several surfactant metabolism related pathways, which are critical for AT2 cells, have been enriched from the up-regulated genes in GI, indicating that GI might retain part of normal lung cell functions and could be considered as early-stage tumors. We did realize that the sentence can easily lead to confusion, and we have refined the text to “Conversely, a number of genes involved in maintenance of normal cell functions and metabolic processes were significantly downregulated from GI to GII (Figure 3B).” (Page 9).

In addition, we have performed differential H3K27ac analyses between GI and normal samples as well as between GII and normal samples (Figure R3-A, C). We then associated the identified differential H3K27ac peaks with genes and assessed the enrichment for oncogenic signatures (from MSigDB Oncogenic Signatures) among the genes. As we can see, both GI up-regulated genes and GII up-regulated genes have been enriched with oncogenic signatures, while no gene sets of oncogenic signatures have been enriched from the up-regulated genes in normal samples (adjusted p-value<0.01; Figure R3-B, D). Note also that there were some oncogenic signatures common to GI and GII (Figure R3-E). Altogether, we believe there were indeed some dysfunctional oncogenic pathways in GI.

**References:**

1.	Cheung WKC, Nguyen DX: **Lineage factors and differentiation states in lung cancer progression.** *Oncogene* 2015, **34:**5771-5780.

2.	Choi J, Park JE, Tsagkogeorga G, Yanagita M, Koo BK, Han N, Lee JH: **Inflammatory Signals Induce AT2 Cell-Derived Damage-Associated Transient Progenitors that Mediate Alveolar Regeneration.** *Cell Stem Cell* 2020, **27:**366-+.

**Q12.** *Figures 4A and 4B need to be adjusted to make them more clear. Besides, what's the meaning of 'ER' in these figures? It is very confusing.*

**Response 12:**

We apologize for the confusion. We have adjusted Figure 4A and Figure 4B to demonstrate the co-expression networks more clearly in the revised manuscript (Figure 4-A, B). We also replaced the abbreviation ‘ER’ with ‘epigenetic regulator’ in Figure 4A. Epigenetic regulators are those genes involved in regulation of epigenetic modifications, including genes related to DNA methylation, histone modification, and chromatin remodeling [1]. For example, EZH2 and KMT2B, two histone methyltransferases defined as GII-specific epigenetic regulators in our study, are involved in the deposition of H3K27me3 and H3K4me3, respectively [2]. For the analysis, we have obtained a list of epigenetic regulators from dbEM [3] and have selected those regulators that were differentially expressed between GI and GII.

**References:**

1.	Dawson MA, Kouzarides T: **Cancer Epigenetics: From Mechanism to Therapy.** *Cell* 2012, **150:**12-27.

2.	Lue JK, Amengual JE: **Emerging EZH2 Inhibitors and Their Application in Lymphoma.** *Current Hematologic Malignancy Reports* 2018, **13:**369-382.

3.	Nanda JS, Kumar R, Raghava GPS: **dbEM: A database of epigenetic modifiers curated from cancerous and normal genomes.** *Scientific Reports* 2016, **6**.

**Q13.** *In my opinion, the authors think distal enhancers and SEs are the same according to Figures 5A and 5B?*

**Response 13:**

We apologize for the misleading text. Distal enhancers were defined as those H3K27ac peaks that were located >2 kb away from all annotated transcription start sites. Super-enhancers (SEs) are clusters of adjacent enhancers with unusually high activity. We identified SEs for each H3K27ac ChIP-seq sample by using ROSE [1, 2], which was a software specifically developed for identifying SEs. Thus, distal enhancers are different from SEs. In the revised manuscript, we used the term “epigenetic regulatory elements” to include both distal enhancers and SEs (Figure 5-A).

**References:**

1.	Whyte WA, Orlando DA, Hnisz D, Abraham BJ, Lin CY, Kagey MH, Rahl PB, Lee TI, Young RA: **Master Transcription Factors and Mediator Establish Super-Enhancers at Key Cell Identity Genes.** *Cell* 2013, **153:**307-319.

2.	Loven J, Hoke HA, Lin CY, Lau A, Orlando DA, Vakoc CR, Bradner JE, Lee TI, Young RA: **Selective Inhibition of Tumor Oncogenes by Disruption of Super-Enhancers.** *Cell* 2013, **153:**320-334.

**Q14.** *What is the definition of core-regulators? How to get those core-regulators?*

**Response 14:**

Thanks for pointing out this issue. In the revised manuscript, we provided more details about the definition of core-regulators and how to get them (Page 11, 17, 18). Briefly, we aimed at selecting those regulators as core-regulators that formed a highly internally connected clique and were associated with as many other regulators in the co-expression network (Figure 4-B) as possible. For this purpose, we involved two important definitions: “regulator score” of each regulator was defined as the number of cliques in which the regulator participated; “clique score” of each clique in the network was defined as the sum of regulator scores of all regulators in the clique. Based on these definitions, we first calculated the regulator score of each regulator in the co-expression network, then calculated the clique score of each clique in the network, and finally selected the regulators of the clique with the highest clique score as core-regulators.

**Q15.** *In line 4 of the Method section, did the authors consider the "other effects" in the DESeq2 model? Please clarify the method of how to identify DEGs from the RNA-seq profiles of LUAD patients.*

**Response 15:**

Sincerely thanks for this suggestion. In the previous differential analysis, DESeq2 was invoked with the design formula “~group”, where group had two levels (i.e., “GI” and “GII”). DEGs were defined as those genes with an adjusted p-value less than 0.05. Following the reviewer’s suggestion, in the revised manuscript we added a covariate “gender” to the design formula, which had levels “male” and “female”. We then defined DEGs based on the same cutoff and re-performed the associated downstream analyses. In the revised manuscript, we have given a detailed description of the differential RNA-seq analysis in the Methods section (Page 17), and have updated all related results. But note that the DEG lists resulting from the two designs were very similar to each other (Figure R4).

**Q16.** *I suggest adding tumor purity estimation of the LUAD samples, as it seems like that the enriched pathways got from the H3K27ac data are very different from the RNA-seq data.*

**Response 16:**

Sincerely thanks for this suggestion. Following this suggestion, we have estimated the tumor purity of the LUAD samples by using hematoxylin-eosin staining (HE staining), and we found the majority of the samples had a tumor purity larger than 70% (Supplementary Table 1). For the differences in enriched pathways between H3K27ac and RNA-seq analyses, we believe the primary reason is that the differential H3K27ac analysis was between tumor and normal lung tissues while the differential RNA-seq analysis was between GI and GII tumors.

To directly assess the consistency between H3K27ac and RNA-seq data, we performed a differential H3K27ac analysis between GI and GII as well and associated the identified differential H3K27ac peaks with their target genes. We then performed pathway enrichment analysis using separately the “DEGs” from H3K27ac and RNA-seq data (the gene sets of KEGG pathways were obtained from the Molecular Signatures Database (MSigDB)). For illustration, Figure R5-A, B gives the top 10 significantly enriched pathways for each enrichment analysis. We found that there were several common pathways between H3K27ac and RNA-seq data (e.g., ‘Cell cycle’ and ‘Valine, leucineand isoleucine degradation’; Figure R5-C, D). Therefore, we think the H3K27ac data are generally consistent with the RNA-seq data.

**Q17.** *"This finding emphasizes that SE abnormalities might be crucial factors in tumorigenesis." Experiments such as pathological staining need to be done to show if those super-enhancers or SE associated transcription factors are correlated with LUAD tumorigenesis or progression.*

**Response 17:**

Sincerely thanks for this valuable suggestion. Following this suggestion, we first performed IHC staining of SOX9, which was associated with differential SEs between normal and tumor tissues. The differential SEs were more active in tumors (Figure 1-E, H), and SOX9 consistently showed clearly higher expression levels in tumor tissues than in normal ones (Figure 1-F). We then selected two transcription factors (*RUNX2* and *NKX2-1*) that were associated with differential SEs between GI and GII tumors (Figure 3-D), and IHC staining experiments for them have showed consistent differential expression patterns (Supplementary Figure 7-C, D). Note also that *NKX2-1* was associated with a decreased expression level in the more malignant GII (compared to the expression level in GI), which is consistent with previous studies showing that *NKX2-1* can suppress LUAD progression [1].

**References:**

1.	Winslow MM, Dayton TL, Verhaak RGW, Kim-Kiselak C, Snyder EL, Feldser DM, Hubbard DD, DuPage MJ, Whittaker CA, Hoersch S, et al: **Suppression of lung adenocarcinoma progression by Nkx2-1.** *Nature* 2011, **473:**101-U120.

**Q18.** *'Based on the literature and RNA-seq data, we noticed CLU and PLA2G1B, two GI-specific genes which were both included in our previous TSGs screening study.' I am still not sure why the authors select CLU as the top-ranked TSG and validate it in-vivo, the rationale needs to be clarified. Would the authors rank all the TSG candidates?*

**Response 18:**

Thanks for pointing out this issue. In the revised manuscript, we have added a detailed description of the rationale behind the selection of *CLU* (Page 12). Specifically, we first noticed that several target genes of GI core regulators (in the network shown by the right panel of Figure 4-A) were well-known TSGs of LUAD, such as *DLC1* (Figure 5-A, left panel). For a systematic evaluation of the enrichment of TSGs among target genes of GI core regulators, we collected a list of 49 highly reliable TSGs, whose expression was consistently down-regulated in 11 different cancer types [1]. We then found that 7 of the TSGs were target genes of GI core regulators, contrasting with that none of the TSGs were target genes of GII core regulators (Figure 5-A, right panel). Besides, most of the TSGs were positively correlated with GI core regulators but negatively correlated with GII core regulators (Supplementary Figure 10-A). Altogether, these observations suggested the possibility of finding new LUAD TSGs among the target genes of GI core regulators. Following this direction, we took the target genes of three GI core regulators (*HLF, POU2F3, KLF15*) that were regulated by super enhancers (Figure 5-B), and selected those genes that were differentially expressed between GI-like and GII-like patients (from the TCGA-LUAD cohort) but did not have significantly differential expression between normal tissues and GI-like tumor tissues (Figure 5-C; Figure R6), as we think such genes might be more likely to be relevant to the progression of LUAD. As a result, we got three TSG candidates in total, which were ACADSB, ABCC6 and CLU. Considering the TSG screening results in our previous study [2], we finally selected CLU for experimental validation (Figure 5E-J).

**References:**

1.	Zhao M, Kim P, Mitra R, Zhao JF, Zhao ZM: **TSGene 2.0: an updated literature-based knowledgebase for tumor suppressor genes.** *Nucleic Acids Research* 2016, **44:**D1023-D1031.

2.	Wu QB, Tian YH, Zhang J, Tong XY, Huang H, Li S, Zhao H, Tang Y, Yuan CZ, Wang K, et al: **In vivo CRISPR screening unveils histone demethylase UTX as an important epigenetic regulator in lung tumorigenesis.** *Proceedings of the National Academy of Sciences of the United States of America* 2018, **115:**E3978-E3986.

**Q19.** *In Figures 5D and 5E, the authors showed the mouse model results. Knocking out CLU increased the lung tumor burden in tumor recipient mice. However, did the authors inject the group I patient-derived tumor cells into the recipient mice? Why the recipient mouse develop the group I type LUAD? H&E, Ki-67, and CLU staining should be added to convince the role of CLU as a tumor suppressor in Group I LUAD.*

**Response 19:**

Sincerely apologize for the misleading text in the original manuscript. We were actually *not* specifically aimed at proving that *CLU* is a GI-LUAD specific tumor suppressor, but wanted to verify a general role of it as a LUAD suppressor. For the mouse experiment, instead of collecting and injecting patient-derived tumor cells into the recipient mice, we directly knocked out *CLU* in K-mouse (KrasG12D/+) via nasal inhalation [1]. We observed significantly higher tumor burden in *CLU* knock-out K-mouse than that in wild-type K-mouse, indicating *CLU* may function as a tumor suppressor during LUAD progression. We also performed experiments on cell lines to investigate the role of *CLU* (Figure 5 E-J) and, as suggested, conducted *CLU* staining as well as *Ki-67* and HE staining (Figure 5-K, L). The expression level of *CLU* was clearly negatively correlated with *Ki-67* expression level, and higher *CLU* expression levels were observed in GI samples than in GII samples. These results indicated a general role of *CLU* as a LUAD suppressor (a more detailed description of the results can be found in the revised manuscript; Page 12).

**References:**

1.	Wu QB, Tian YH, Zhang J, Tong XY, Huang H, Li S, Zhao H, Tang Y, Yuan CZ, Wang K, et al: **In vivo CRISPR screening unveils histone demethylase UTX as an important epigenetic regulator in lung tumorigenesis.** *Proceedings of the National Academy of Sciences of the United States of America* 2018, **115:**E3978-E3986.

**Q20.** *In line 53 of the Discussion section. The authors concluded that GII is transformed from GI at an early stage. How did they get the temporal relationship between GII and GI? Did they conduct trajectory analysis to demonstrate that? More late-stage samples in GII than GI doesn't mean that GII and GI have the same tumor progenitors.*

**Response 20:**

We very appreciate this comment. We indeed have not performed trajectory analysis to prove the temporal relationship between GI and GII. Results of several analyses in this study, however, have led us to the speculation that GII might be transformed from GI. For example, compared to GI, we observed the loss of expression of several LUAD differentiation markers (e.g., *NKX2-1*), down-regulated activity of lineage identity related pathways (e.g., surfactant metabolism related pathways), and several inactivated TSGs (such as *DLC1*) in GII (Figure 3-B, D; Supplementary Figure 7-C; Figure 5-A). Besides, in the differential RNA-seq analysis between GI and GII, basically no oncogenic pathways have been enriched from the identified differential genes that were more highly expressed in GI (Figure 3-A, B), suggesting GI might precede GII along the timeline of LUAD progression.

We do agree that we lack direct evidences at present for proving the temporal relationship between GI and GII, and we have modified the associated text about this hypothesis in the revised manuscript (Page 14). For future studies, we may conduct direct tracer experiments to explore this hypothesis.

**Minor Comments:**

**Q1.** *Abbreviations need to be spelled out or defined when they first appear. For example, PFS and OS.*

**Response 1:**

Sincerely thanks for pointing out this issue. We have accordingly corrected the text in the revised manuscript (Page 9).

**Q2.** *There are many syntax errors. For example, in line 52, 'By contrast, the tumor-specific sites are highly enriched in transcriptional regulation, indicating that aberrant epigenetic modification serves dysregulate transcription in LUAD (Figure 1-D).'; 'These data suggest that SE abnormality directly get involved in LUAD oncogenesis by regulating driver genes. Taken together, the results underscored the role of SE in LUAD as key regulators.'; 'When perform survival analysis in each stage, we found that GI-GII score can identify high-risk patients in stage I cohort (Figure 3-H).'; 'Differential epigenetic element acted as an upstream influenced the expression of these core regulators.'*

**Response 2:**

We apologize for the many syntax errors in the original manuscript. In the revised manuscript, we carefully checked the text and tried our best to correct syntax errors.

**Reviewer #2:** **Overall Summary:**

*In this study, Yuan et al. applied the histone modification H3K27ac to facilitate the tumor-subtyping of lung adenocarcinoma. First, the authors showed the global H3K27ac profile in tumor and normal samples, and the differential peaks are related to transcription dysregulation in LUAD. The super enhancer-associated genes are also related to oncogenesis. In addition, fusion gene expression is correlated with abnormality of super enhancer activity. Then they quantified the variation of H3K27ac signals in tumor samples, and termed it as the 'intertumor epigenomic heterogeneity'. Two LUAD subgroups (GI and GII) were classified using the most variable H3K27ac peaks, and they have distinct pathological risk level and survival rate. Based on the subgroup specific genes, they reanalyzed LUAD data in TCGA cohort and defined GI-like and GII-like patients. And the survival rate and mutation frequency of these two groups of patients are different. Furthermore, the regulating networks of GI and GII-specific genes are distinct. Finally, the authors came up with a candidate gene, CLU, which is lower expressed in GII but not GI. And the knock-out experiments suggest it may serve as a TSG. Overall, this study expanded our understanding of tumor heterogeneity from the perspective of histone modification. But some description and definitions are not clearly presented. Before considered as suitable for publication, some concerns need to be clarified first.*

**Response in summary：**

We thank this reviewer for her/his critical and insightful comments. In the revision, we have made changes to solve the corresponding concerns. We modified figures and figure legends as suggested. We also provided more detailed information about cutoff threshold and PC percentage of each PC in our revised manuscript. We added detailed description about how GII was further dived into GII.1 and GII.2. The detailed responses were listed below. We hope this reviewer will find the manuscript much improved.

**Response to the comments:**

**Reviewer #2**

**Q1.** *Recently, two Cell papers revealed the molecular signatures of lung cancer mainly based on proteomics. What's the benefit of H3K27ac-based subtyping compared to RNA or proteomic subtyping? Whether it can better explain survival or pathological risk?*

**Response 1:**

Sincerely thanks for pointing out this issue. LUAD is a disease with high heterogeneity and it is important to understand the biological processes and features during tumorigenesis and progression. Previous studies have proposed different classification models based on genomics or transcriptomics [1,2,3]. Recent studies revealed molecular signatures during LUAD tumorigenesis and progression based on proteomics [4,5]. These models deepen our understanding of LUAD and help us predict patients’ prognosis and determine individual treatments. Our H3K27ac-based classification can also well predict the survival or pathological risk, and it can provide additional information about epigenetic regulations compared to the previous models. Here, we performed a comparison between H3K27ac-based subtyping and RNA-seq based subtyping by using our LUAD cohort (Figure 2-A, D; Supplementary Figure 5-A, B, C). We first called hyper-variable genes in RNA-seq data using Limma-Trend [6] (Supplementary Figure 5-B) and then performed principle component analysis (PCA) and hierarchical clustering based on PC1 as in the previous analysis (Supplementary Figure 5-C). On one hand, two classification results were similar to each other (Supplementary Figure 5-D). On the other hand, the clustering results based on H3K27ac ChIP-seq data are more stable compared to RNA-seq based clustering results (Supplementary Figure 5-A, C). Besides, the prognosis differences between subtypes are more significant in the H3K27ac subtyping than in the RNA-seq subtyping (Supplementary Figure 5-E). In summary, these two subtyping methods can both well distinguish between good and poor survival patients, and the H3K27ac subtyping can relatively better explain the survival differences among patients than the RNA-seq subtyping.

**References:**

1.	Beer DG, Kardia SLR, Huang CC, Giordano TJ, Levin AM, Misek DE, Lin L, Chen GA, Gharib TG, Thomas DG, et al: **Gene-expression profiles predict survival of patients with lung adenocarcinoma.** *Nature Medicine* 2002, **8:**816-824.

2.	Bhattacharjee A, Richards WG, Staunton J, Li C, Monti S, Vasa P, Ladd C, Beheshti J, Bueno R, Gillette M, et al: **Classification of human lung carcinomas by mRNA expression profiling reveals distinct adenocarcinoma subclasses.** *Proceedings of the National Academy of Sciences of the United States of America* 2001, **98:**13790-13795.

3.	Network CGAR: **Comprehensive molecular profiling of lung adenocarcinoma (vol 511, pg 543, 2014).** *Nature* 2018, **559**.

4.	Chen YJ, Roumeliotis TI, Chang YH, Chen CT, Han CL, Lin MH, Chen HW, Chang GC, Chang YL, Wu CT, et al: **Proteogenomics of Non-smoking Lung Cancer in East Asia Delineates Molecular Signatures of Pathogenesis and Progression.** *Cell* 2020, **182:**226-+.

5.	Gillette MA, Satpathy S, Cao S, Dhanasekaran SM, Vasaikar SV, Krug K, Petralia F, Li YZ, Liang WW, Reva B, et al: **Proteogenomic Characterization Reveals Therapeutic Vulnerabilities in Lung Adenocarcinoma.** *Cell* 2020, **182:**200-+.

6.       Law CW, Chen YS, Shi W, Smyth GK: **voom: precision weights unlock linear model analysis tools for RNA-seq read counts**. *Genome Biology* 2014, **15**.

**Q2.** *The legend of Fig1A is not correct. The clustering is based on the Pearson's correlation coefficient (PCC), but not H3K27ac signals.*

**Response 2:**

Sincerely thanks for pointing out this issue. In the revised manuscript, we have corrected the legend of Figure1A to “Unsupervised hierarchical clustering of H3K27ac profiles for tumor and normal lung tissues of LUAD patients based on pairwise Pearson correlation coefficients (PCCs).”.

**Q3.** *What does the H3K27ac profile at ALK and EML4 look like in ALK-EML4 fusion samples? Any difference from non-fusion samples?*

**Response 3:**

We thank reviewer for this suggestion. We have adjusted the Figure 1H (Figure 1-I in revised manuscript) in the new version manuscript. The H3K27ac profile at *ALK* and *EML* loci were similar in *EML4-ALK* fusion sample and non-fusion sample (Figure R7), however, the expression level of *ALK* was increased in fusion sample (Figure 1-I, bottom panel).

**Q4.** *For the PCA analysis, what is the percentage of each PC?*

**Response 4:**

We apologize for the confusing description. In the revised manuscript, we have made a correction. “The percentage of each PC” has been replaced by “Percentage of variance explained by each PC” (Supplementary Figure 4-D).

**Q5.** *The authors showed that H3K27ac-based subtypes present different mutation frequency. Whether the regional mutation rate is associated with H3K27ac variation level?*

**Response 5:**

Sincerely thanks for this valuable suggestion. This is a very good point regarding the potential association between mutation rate and H3K27ac variability. Since we did not have whole genome sequencing data for our LUAD patients (nor did we have H3K27ac ChIP-seq data for the TCGA-LUAD cohort), we used our H3K27ac ChIP-seq data of tumor and normal lung tissues for calling somatic SNVs (using MuTect [1]). To increase the read coverage, we first separately merged tumor and normal ChIP-seq samples, and then identified somatic SNVs of the merged tumor profile against the merged normal profile. Most of the identified somatic SNVs were associated with a coverage larger than 10 reads (Figure R8-A). We found that the total number of somatic SNVs falling within the tumor HVPs (hyper-variable peaks) was significantly greater than the number of somatic SNVs at randomly sampled peaks (random sampling has been performed 1000 times, in each of which we have randomly selected the same number of H3K27ac peaks as the number of tumor HVPs; Figure R8-B). This observation suggested that there is certain association between mutation rate and H3K27ac variability.

**References:**

1.	Cibulskis K, Lawrence MS, Carter SL, Sivachenko A, Jaffe D, Sougnez C, Gabriel S, Meyerson M, Lander ES, Getz G: **Sensitive detection of somatic point mutations in impure and heterogeneous cancer samples.** *Nature Biotechnology* 2013, **31:**213-219.

**Q6.** *The authors should explain how the GII group was further divided to GII.1 and GII.2. I cannot find any description or threshold.*

**Response 6:**

We thank reviewer for pointing this out. We further divided GII tumor samples into GII.1 and GII.2 based on previous hierarchical clustering result (Supplementary Figure 7-E). We have added more details in the revised manuscript (Page 9).

**Q7.** *The definition of GI-like and GII-like patients are based on similarity of transcriptomes. But gene expression is not fully determined by H3K27ac, so such classification may or may not related to H3K27ac profile.*

**Response 7:**

Sincerely thanks for pointing out this issue. We found that the classification results based on H3K27ac ChIP-seq data and the classification results based on RNA-seq data were very similar (please see Q1; Supplementary Figure 5-D). Besides, for practical consideration, there are more available RNA-seq data sets than H3K27ac ChIP-seq data sets (e.g., the former is available for the TCGA-LUAD cohort while the latter is not). In order to make our classification model more widely applicable, we have designed a classifier based on RNA-seq profiles.

We used GI and GII gene signatures to evaluate the similarity of each RNA-seq sample to GI/GII. Specifically, we calculated two ssGSEA scores for each tumor sample [1]. In this way, GI and GII were well separated from each other (Figure R9). Thus, our classification method could well distinguish between GI and GII based on RNA-seq data. Applying this method to the TCGA-LUAD RNA-seq data set, we got an acceptable classification result and divided the samples into high-risk and low-risk ones (Figure 3-G, H).

**References:**

1.	Barbie DA, Tamayo P, Boehm JS, Kim SY, Moody SE, Dunn IF, Schinzel AC, Sandy P, Meylan E, Scholl C, et al: **Systematic RNA interference reveals that oncogenic KRAS-driven cancers require TBK1.** *Nature* 2009, **462:**108-U122.

**Q8.** *The authors claimed 'higher epigenomic heterogeneity in GII than GI'. But they haven't specify which part of results can support it.*

**Response 8:**

We thank reviewer for pointing this out. We have added new analysis to support it. We used the H3K27ac data to calculate the sample-wise pearson correlation coefficient (PCC) within GI and GII respectively. These PCCs in GI were significantly larger than those in GII (Figure R10). Thus, we observed higher epigenomic heterogeneity in GII than GI

**Q9.** *The figures and legends need re-organization. Some of them are not explained in main text, like Fig1G. And I don't know why the 'chrY' are highlighted in Fig2A. And the order of some figures are not consistent with text.*

**Response 9:**

Sincerely thanks for this suggestion. In the revised manuscript, we have re-organized the figures and the references to figures in the main text. As for the highlighting of ‘chrY’, it is because we observed a group of special peaks that looked somewhat separated from the other peaks (Figure R11-A). These peaks tended to have small mean signal intensities and relatively large variances. It turned out that these peaks were largely located in chromosome Y. We therefore specifically highlighted the peaks in chromosome Y in the original Figure 2A (Figure R11-B). We agree that this highlighting was not very relevant to the main point of the figure, and we removed the ‘chrY’ labels in the revised manuscript.

**Second round of review**

**Reviewer 1**

Overall Summary:

Although the authors answered all my questions, most of the responses still are unconvincing. The authors should also validate important super enhancers for different subgroups in vivo because super enhancers are directly identified from the H3K27Ac data. In addition, not sure any of the master transcription factors identified from the H3K27AC ChIP-seq analysis, such as SOX9 and CLU implicate lung cancer cellular origins. Furthermore, the experiments can’t support the H3K27Ac ChIP-seq analysis results, for example, CLU is predicted as a GI-LUAD specific tumor suppressor, but the authors only validated CLU in a general lung cancer mouse model but not a GI-LUAD specific mouse model. I have more questions detailed below.

Major Comments:

1. On Page6 line37-line38: Not sure what long-range enhancer-promoter loops mean. In this paragraph, the topic is about tumor/normal specific SE target genes, not core regulatory networks. Do enhancer-promoter loops mean that the target genes of specific SEs could express and target back to these specific enhancers?

2. Why pick up Sox9 for H&E, the authors cherry-picked up Sox9 for testing. Could the authors rank those SE associated transcription factors and pick up the top-ranked ones for testing?

3. Again, Fig 1G listed out a lot of enriched pathways or GO terms, not sure any of those GO terms or pathways are correlated with LUAD tumorigenesis or progression. These results are misleading. Also, Fig3B, the Go terms or pathway enrichment analysis didn’t point out the important oncogenic pathways.

4. Page 8 line 12: Could the authors please explain why cluster the patients by the PC1 score? For my understanding, the PC1 score reflects the ‘weights’ of each sample in PC1 that is the phenotype lymph node invasion to which the PC1 score is strongly related in this paper. So, when you clustered samples by PC1, you were actually clustering the samples by the lymph node invasion level because it is highly correlated with the PC1 score, which makes a significant difference in lymph node invasion in G1 and G2 as a certain correlation.

5. Not sure what degree means in Figure4C. Did the authors calculate the inward and outward degree from Core regulatory network?

6. The average signal of enhancers within a SE would not be a good way to measure the SE signal. As larger SEs contained more enhancers could be more important than the smaller one. The average signal might diminish the real significance of large super enhancers.

7. Not sure any of the master transcription factors identified from the H3K27AC ChIP-seq analysis, such as SOX9 and CLU implicate lung cancer cellular origins.

8. The authors should also validate important super enhancers for different subgroups in vivo because super enhancers are directly identified from the H3K27Ac data.

9. CLU is predicted as a GI-LUAD specific tumor suppressor, but the authors only validated CLU in a general lung cancer mouse model but not a GI-LUAD specific mouse model.

**Reviewer 2**

The authors have adequately addressed my concerns.

**Authors Response**

**Point-by-point responses to the reviewers’ comments:**

**Reviewer #1: Overall Summary:**

Although the authors answered all my questions, most of the responses still are unconvincing. The authors should also validate important super enhancers for different subgroups in vivo because super enhancers are directly identified from the H3K27Ac data. In addition, not sure any of the master transcription factors identified from the H3K27AC ChIP-seq analysis, such as SOX9 and CLU implicate lung cancer cellular origins. Furthermore, the experiments can’t support the H3K27Ac ChIP-seq analysis results, for example, CLU is predicted as a GI-LUAD specific tumor suppressor, but the authors only validated CLU in a general lung cancer mouse model but not a GI-LUAD specific mouse model. I have more questions detailed below.

**1.** *On Page6 line37-line38: Not sure what long-range enhancer-promoter loops mean. In this paragraph, the topic is about tumor/normal specific SE target genes, not core regulatory networks. Do enhancer-promoter loops mean that the target genes of specific SEs could express and target back to these specific enhancers?*

**Response: Thanks for pointing this out. “long-range enhancer-promoter loops” in the original manuscript refers simply to long-distance enhancer-promoter interactions through chromatin looping [1]. We agree that phrase may lead to confusion and has substituted “enhancer-promoter interactions” for “enhancer-promoter loops” in the revised manuscript (Page 6).**

**References:**

**[1]** Whalen S, Truty RM, Pollard KS: **Enhancer-promoter interactions are encoded by complex genomic signatures on looping chromatin.** Nat Genet 2016, 48:488-496.

**2.** *Why pick up Sox9 for H&E, the authors cherry-picked up Sox9 for testing. Could the authors rank those SE associated transcription factors and pick up the top-ranked ones for testing?*

**Response: Thanks for this suggestion. We have ranked all transcription factors (TFs) associated with the tumor-specific super-enhancers, according to the testing *p*-values from the differential super-enhancer analysis between normal and tumor tissues (please see Methods, page 16). As shown in Figure 1E, SOX9 is ranked as the number one TF. We additionally performed IHC staining for other top-ranked TFs, including *SIX1*, *RUNX1*, and *SOX4* (Figure 1E). Our data also showed an increased immunostaining of these TFs in tumor tissues in comparison with normal tissues (Supplementary Figure 3E).**

**References:**

**[1]** Laughney AM, Hu J, Campbell NR, Bakhoum SF, Setty M, Lavallee VP, Xie Y, Masilionis I, Carr AJ, Kottapalli S, et al: **Regenerative lineages and immune-mediated pruning in lung cancer metastasis.** Nat Med 2020, 26:259-269.

**3.** *Again, Fig 1G listed out a lot of enriched pathways or GO terms, not sure any of those GO terms or pathways are correlated with LUAD tumorigenesis or progression. These results are misleading. Also, Fig3B, the Go terms or pathway enrichment analysis didn’t point out the important oncogenic pathways.*

**Response: Thanks for this comment. For Figure 1G, we listed in Table R1 many of the pathways enriched by the genes associated with tumor-specific super-enhancers that have been reported to be related to LUAD tumorigenesis or progression in previous studies. In particular, some of these pathways are related to cancer stem cell (CSC) or LUAD cancer cell migration and invasion, including chordate embryonic development, cell fate specification, regulation of cell adhesion, and collagen catabolic process. More specifically, these pathways are involved in EMT (epithelial to mesenchymal transition) and extra-cellular matrix organization, which are key processes for cancer cell migration and invasion. Besides, as we have mentioned in the previous response letter, the activity of these pathways is significantly correlated with the lymph node invasion status (please see Q3 in the previous response letter).**

**Table R1**

**For Figure 3B, we listed several pathways enriched by the DEGs between GI and GII (Table R2). These pathways have been found to be related to oncogenic events in previous LUAD studies. Specifically, GI-up regulated genes are enriched with those genes involved in complement and coagulation cascades, inorganic ion homeostasis, and surfactant homeostasis, all of which have been found to be associated with an increased activity in *KRAS*-mutant LUAD patients. As for GII-up regulated genes, they are primarily enriched in pathways related to cell cycle progression and DNA replication, both of which are signatures of highly malignant tumors. Besides, the** ***FOXM1* pathway** **has been proven to be related to LUAD invasion and metastasis, which is consistent with the fact that more patients in the GII group are associated with lymph node invasion. The** ***E2F* pathway regulates a wide range of biological processes, including cell cycle, apoptosis, and DNA damage response. Over-activation of this pathway could lead to tumorigenesis and poor prognosis in non-small cell lung carcinoma.**

**We have added a more detailed explanation of the enriched pathways and have cited the corresponding published evidence in the revised manuscript (Page 7 and 9).**

**Table R2**

**4.** *Page 8 line 12: Could the authors please explain why cluster the patients by the PC1 score? For my understanding, the PC1 score reflects the ‘weights’ of each sample in PC1 that is the phenotype lymph node invasion to which the PC1 score is strongly related in this paper. So, when you clustered samples by PC1, you were actually clustering the samples by the lymph node invasion level because it is highly correlated with the PC1 score, which makes a significant difference in lymph node invasion in G1 and G2 as a certain correlation.*

**Response: Thanks for pointing this out, but we believe there is no problem associated with the principle component analysis (PCA) and the clustering analysis of the PC1 scores. The PCA was purely based on the hyper-variable peaks identified from the H3K27ac ChIP-seq data, which were independent of the meta data regarding the lymph node invasion states of patients. The strong correlation between PC1 and lymph node invasion status was simply an observation on the PCA results but did not result from computational artefacts. The only reason for which we used only PC1 for the clustering analysis was that the PC1 alone has explained a large proportion (32.3%) of the total variance, and this proportion was much larger than explained by PC2 (7.8%).**

**Besides, the lymph node invasion status was not exactly equivalent to PC1. Here we also tried clustering the patients based on their lymph node invasion status. The resulting classification into two groups was different from that based on PC1, and our PC1-based classification was associated with a more significant survival difference (Figure R1A, B). Furthermore, we tried using different numbers of top PCs (up to PC5) to cluster the patients, and all the resulting two-group classifications were exactly the same (Figure R1C, D).**

**5.** *Not sure what degree means in Figure4C. Did the authors calculate the inward and outward degree from Core regulatory network?*

**Response: We apologize for the confusion. The “Degree” in Figure 4C is calculated from the bipartite networks shown in Figure 4A. In each of the bipartite networks, all the involved genes can be split into two subsets, corresponding respectively to regulators (including transcription factors and epigenetic regulators) and non-regulator genes, and each link connects a regulator to a non-regulator gene (Figure R2A) if there is a significant co-expression. Thus, all the links are essentially undirected. Therefore, the “Degree” in Figure 4C indicates the number of non-regulator genes associated with each GI/GII-specific regulator, and we found that the degrees of the core regulators were significantly larger than those of the other regulators (Figure R2B). We have added more details in the figure legend and the plot in the revised manuscript.**

**6.** *The average signal of enhancers within a SE would not be a good way to measure the SE signal. As larger SEs contained more enhancers could be more important than the smaller one. The average signal might diminish the real significance of large super enhancers.*

**Response: Thanks for pointing this out. This is a good point regarding the variation of size across SEs, and we agree that the size of a specific SE reflects its importance. But we must clarify that we did not use the average signal of enhancers within each individual SE for the identification of differential SEs. The average signals have only been used for visualization (Figure 1D; converted into z-scores) as well as for assessing the correlations between SE signals and promoter H3K27ac signals or gene expression (see Methods; page 16).**

**For the differential SE analysis, we first used MAnorm2 to identify differential H3K27ac peaks [1]. We then used the Fisher-exact test to assess the enrichment of differential enhancers (i.e. distal H3K27ac peaks) within each SE. After multiple testing adjustment, SEs with an adjusted *p*-value less than 0.05 were defined as differential SEs (see Methods and Supplementary Figure 2; page 16). This method for differential SE analysis has taken the number of enhancers within each SE into account, and it can be seen that the method tends to assign smaller *p*-values to SEs containing more enhancers (Figure R3A). Besides, since the enhancers within each individual differential SE show very consistent signal changes across conditions (Figure R3B), we think that using the average signal of the enhancers is suitable for visualizing the variation of the SE activity across samples.**

**References:**

**[1]** Tu S, Li M, Chen H, Tan F, Xu J, Waxman DJ, Zhang Y, Shao Z: **MAnorm2 for quantitatively comparing groups of ChIP-seq samples.** Genome Res 2021, 31:131-145.

**7.** *Not sure any of the master transcription factors identified from the H3K27AC ChIP-seq analysis, such as SOX9 and CLU implicate lung cancer cellular origins.*

**Response: Thanks for this comment. In our previous TSG screening study, we highlighted the potential role of *CLU* as a TSG in an LUAD mouse model [1]. As for *SOX9*, it has been found to be a key regulator in LUAD progression and EMT in multiple previous studies [2,3] as well as a lung-specifying transcription factors. A recent study found that *SOX9* could characterize primary lung adenocarcinomas participating in primitive transcriptional programs spanning stem-like to regenerative pulmonary epithelial progenitor states. *SOX9* acts as a regulator of proliferation and differentiation in epithelial stem/progenitor cells and involves in the lung epithelium during branching morphogenesis [4]. Previous study also found SOX9 expression was essential for latency competent cancer cells invading NK-cell-mediated immune surveillance in early stage lung cancer [5]. Moreover, both of the two genes are of prognostic value for LUAD patients. Specifically, we performed survival analyses by dividing a cohort of LUAD patients [6] into two equally-sized groups based on the expression levels of *CLU* or *SOX9*, and we observed significant survival differences between groups for both genes (Figure R4).**

**References:**

**[1]** Wu QB, Tian YH, Zhang J, Tong XY, Huang H, Li S, Zhao H, Tang Y, Yuan CZ, Wang K, et al: **In vivo CRISPR screening unveils histone demethylase UTX as an important epigenetic regulator in lung tumorigenesis.** Proceedings of the National Academy of Sciences of the United States of America 2018, 115:E3978-E3986.

**[2]** Zhou CH, Ye LP, Ye SX, Li Y, Zhang XY, Xu XY, Gong LY: **Clinical significance of SOX9 in human non-small cell lung cancer progression and overall patient survival.** J Exp Clin Cancer Res 2012, 31:18.

**[3]** Huang JQ, Wei FK, Xu XL, Ye SX, Song JW, Ding PK, Zhu J, Li HF, Luo XP, Gong H, et al: **SOX9 drives the epithelial-mesenchymal transition in non-small-cell lung cancer through the Wnt/beta-catenin pathway.** J Transl Med 2019, 17:143.

**[4]** Laughney Ashley M., Hu Jing., Campbell Nathaniel R., Bakhoum Samuel F., Setty Manu., Lavallée Vincent-Philippe., Xie Yubin., Masilionis Ignas., Carr Ambrose J., Kottapalli Sanjay., Allaj Viola., Mattar Marissa., Rekhtman Natasha., Xavier Joao B., Mazutis Linas., Poirier John T., Rudin Charles M., Pe'er Dana., Massagué Joan.(2020). **Regenerative lineages and immune-mediated pruning in lung cancer metastasis**. Nat Med, 26(2), 259-269. doi:10.1038/s41591-019-0750-6

**[5]** Malladi Srinivas., Macalinao Danilo G., Jin Xin., He Lan., Basnet Harihar., Zou Yilong., de Stanchina Elisa., Massagué Joan.(2016). **Metastatic Latency and Immune Evasion through Autocrine Inhibition of WNT.** Cell, 165(1), 45-60. doi:10.1016/j.cell.2016.02.025

**[6]** Gyorffy B, Surowiak P, Budczies J, Lanczky A: **Online survival analysis software to assess the prognostic value of biomarkers using transcriptomic data in non-small-cell lung cancer.** PLoS One 2013, 8:e82241.

**8.** *The authors should also validate important super enhancers for different subgroups in vivo because super enhancers are directly identified from the H3K27Ac data.*

**Response: Thanks for the suggestion. We identified SEs from H3K27ac data and the directly validation will be helpful to further understand the role of SEs in tumorigenesis. We have planed this part of investigation in our future work because it’s much more difficult to suppress or activate SE activity in vivo than in vitro. And currently there is no GI/GII mouse model available yet. Instead of experiments in vivo, we performed experiments to validate the role of GI-specific enhancers in regulating *CLU* gene expression in vitro.(see our response to comments-9)**

**9.** *CLU is predicted as a GI-LUAD specific tumor suppressor, but the authors only validated CLU in a general lung cancer mouse model but not a GI-LUAD specific mouse model.*

**Response: Thanks for the suggestion. Our study is the first to define the GI-LUAD and currently there is no GI-LUAD mouse model available yet. As suggested, we have now performed more experiments to study the role of GI-specific enhancers in regulating *CLU* gene expression. We designed dual sgRNAs using GPP Web Portal (https://portals.broadinstitute.org/gpp/public/analysis-tools/sgrna-design) to target the predicted enhancer region of *CLU* on chromosome 8: 27469073-27471073, which contains the binding motifs of several TFs (Figure 5M) including *HLF* (a GI core regulator identified in this study), *Nkx2-1* (a GI-specific SE-associated TF) and *TEF* (a GI up-regulated TF compared to GII). We chose 4 top-ranked sgRNAs and then established the dual-sgRNAs (dsgRNAs) constructs as previously described [1]. By PCR analysis, dual-sg2/5 had higher knockout efficiency (Figure 5N). We further confirmed the down-regulation of *CLU* gene expression when the enhancer was knocked out (Figure 5O). These cells also showed significantly increased cell proliferation (Figure 5P). Taken together, our data support the regulation of *CLU* gene expression by GI-specific enhancer, which potentially contributes to inhibiting cancer cell proliferation.**

**References:**

**[1]** Hong Hui., Yao Shun., Zhang Yuanyuan., Ye Yi., Li Cheng., Hu Liang., Sun Yihua., Huang Hsin-Yi., Ji Hongbin.(2020)**. In vivo miRNA knockout screening identifies miR-190b as a novel tumor suppressor.** PLoS Genet, 16(11), e1009168. doi:10.1371/journal.pgen.1009168
